# Characterization and optimization of exopolysaccharide extracted from a newly isolated halotolerant cyanobacterium, *Acaryochloris Al-Azhar MNE ON864448.1* with antiviral activity

**DOI:** 10.1186/s12934-024-02383-4

**Published:** 2024-04-22

**Authors:** Mabroka H. Saad, Nagwa M. Sidkey, Esmail M. El-Fakharany

**Affiliations:** 1 Protein Research Department, Genetic Engineering and Biotechnology Research Institute (GEBRI), City of Scientific Research and Technological Applications (SRTA-City), New Borg El Arab, Alexandria Egypt; 2 Botany & Microbiology Department, Faculty of Science, Al-Azhar University (Girls Branch), Nasr City, Egypt; 3 Pharmaceutical and Fermentation Industries Development Centre (PFIDC), City of Scientific Research and Technological Applications (SRTA-City), New Borg Al Arab, Alexandria Egypt

**Keywords:** Cyanobacterial exopolysaccharide, Plackett–Burman design, Central composite design, Antiviral activity

## Abstract

Several antiviral agents lost their efficacy due to their severe side effects and virus mutations. This study aimed to identify and optimize the conditions for exopolysaccharide (EPS) production from a newly isolated cyanobacterium, *Acaryochloris Al-Azhar MNE ON864448.1*, besides exploring its antiviral activity. The cyanobacterial EPS was purified through DEAE-52 cellulose column with a final yield of 83.75%. Different analysis instruments were applied for EPS identification, including Fourier-transform infrared (FT-IR) spectroscopy, thermogravimetric analysis (TGA), and gas chromatographic-mass spectrometry (GC–MS). Plackett–Burman’s design demonstrated that working volume (X1), EDTA (X2), inoculum size (X3), CaCl_2_ (X4), and NaCl (X5) are the most important variables influencing EPS production. Central composite design (CCD) exhibited maximum EPS yield (9.27 mg/mL) at a working volume of 300 mL in a 1 L volumetric flask, EDTA 0.002 g/L, inoculum size 7%, CaCl_2_ 0.046 g/L, and NaCl 20 g/L were applied. EPS showed potent antiviral activities at different stages of herpes simplex virus type-1 and 2 (HSV-1, HSV-2), adenovirus (ADV) and coxsackievirus (A16) infections. The highest half-maximal inhibitory concentration (IC_50_) (6.477 µg/mL) was recorded during HSV-1 internalization mechanism, while the lowest IC_50_ (0.005669 µg/mL) was recorded during coxsackievirus neutralization mechanism.

**Supplementary Information:**

The online version contains supplementary material available at 10.1186/s12934-024-02383-4.

## Introduction

Viral infections are a serious worldwide problem with considerable morbidity with direct/indirect impacts on the international economy and social life [1]. Multidrug-resistant infections are expected to cause 700,000 mortalities each year [2]. As well, herpes simplex virus (HSV) is a contagious virus and its infection is one of the most prevalent infectious disorders in the community [3]. HSV is classified into two distinct types: HSV type 1 (HSV-1) is primarily transmitted by oral-to-oral contact and causes fever blisters or cold sores around the mouth and on the face but can also cause genital herpes [4], and HSV type 2 (HSV-2) that is sexually transferred and causes genital herpes [5]. According to the World Health Organization (WHO) HSV-1 and HSV-2 are thought to impact roughly 3.7 billion and 491 million persons over the age of 50, respectively [3]. Acyclovir or aciclovir, penciclovir, and valacyclovir are the most common medications for HSV infection [6]. The increase in drug resistance, as well as the challenges of traditional medicines to eliminate latent infections, highlights the need for new exploratory approaches [7]. While there are no effective prevention or mitigation tools, the control of viral infections has serious consequences for social life and the global economy. For example, the recent Coronavirus disease-2019 (COVID-19) pandemic affected all economic areas, education, health treatment, social mobility, and many other human activities [8]. Unfortunately, the effective vaccine strategy is only restricted to certain viral species [9]. As well, developing effective medications is a lengthy and time-consuming procedure that usually fails which is interpreted as the low number of viral medications. In recent years, cyanobacterial exopolysaccharides have received more attention due to their interesting characteristics including their anionic nature, high complexity, and up to six different sugar-repeating units, which is a notable distinction from polymers synthesized by other bacteria or microalgae, which typically have fewer than four sugar repeating units [10].

Focusing on the antiviral activity of cyanobacterial polysaccharides, many studies displayed their potential antiviral activities against various viruses, including both DNA and RNA viruses. In most cases, cyanobacterial polysaccharides exert their inhibiting effect at different viral propagation stages, such as viral adsorption, uncoating, and/or replication steps in host cells. In addition, they exhibit diverse immunomodulatory properties signifying their possible application as vaccine adjuvant [11]*.* Calcium spirulan is a sulfated polysaccharide extracted from *Arthrospira platensis* structurally made up of nine sugars, including mannose, rhamnose, fructose, ribose, galactose, glucose xylose, galacturonic acid, glucuronic acid. This sulfate polysaccharide inhibited virus penetration of HSV-1, human cytomegalovirus (HCMV), measles, mumps, influenza A, and HIV-1 viruses [12] in their host cells. Spirulan-like compounds prevent HSV adsorption and/or penetration as well as obstruct intracellular phases, and viral protein synthesis and consequently suppress viral infection [13]. Reichert et al., [14] reported that EPS from *A. platensis* at concentrations ranging from 18 to 36 g/mL inhibited Koi herpesviruses (KHV) infection. Utilizing the quantitative polymerase chain reaction (PCR) method, Ca-SP inhibitory activity against HSV-1 was found to be highly potent (IC_50_ ranging from 0.05 to 0.5 µg/mL) and comparable to other antiviral agents such as acyclovir [15]. Moreover, an acidic polysaccharide known as nostoflan, extracted from the cyanobacterium *Nostoc flagelliforme*, exhibited potential antiviral activity against different enveloped viruses such as influenza A virus, HCMV, HSV-1, and HSV-2 with IC_50_ values of 78, 0.47, 0.37, and 2.9 g/mL, respectively [16]. Nostoflan exerts its antiviral activity when added at the same time with viruses resulting in blocking the viruses from adsorbing and consequently preventing viral infection [16].

This study aims to contribute to the development of broad-spectrum antiviral agents with different modes of action, fulfilling the urgent need for effective treatments against rapidly mutating viruses. In this investigation, we successfully extracted sulfated exopolysaccharide from newly cyanobacterium isolate; *Acaryochloris Al-Azhar MNE ON864448.1*, using different characterization techniques to elucidate the extracted polysaccharide structure, statistically optimizing its production, and finally exploring its promising antiviral activity against different viruses, including herpes simplex type-1 and 2 (HSV-1, HSV-2), Adenovirus (ADV) and Coxsackievirus (A16). Our study represents a preliminary step to explore a new antiviral agent with a wide spectrum of activity and different modes of action requiring more exploration in terms of antiviral mechanism and in vivo application. Also, the potential antiviral activity of this EPS raises the possibility for future investigations as a carrier for other antiviral drugs or as an adjuvant for vaccines.

## Materials and methods

### Cyanobacterial sampling and isolation

About, 50 water samples are collected from different Egyptian water bodies, including canals, and ponds. The samples were collected in 200 mL sterile jars containing at least 20 mL sterile enriched medium in the daytime (when the cyanobacteria are expected to be available in the photosynthetic area of the water body). Water environmental parameters such as water temperature and pH were recorded to optimize in vitro growth of cyanobacteria [17]. Z^8^ semi-solid medium designed by Kotai, [18] (MgSO_4_.7H_2_O (0.25 g), NaNO_3_ (0.467 g), Ca(NO_3_)_2_·4H_2_O (59 mg), NH_4_Cl (31 mg), Na_2_CO_3_ (0.02 g), Fe-EDTA solution (10 mL), 1 mL of Gaffron micronutrients [(H_3_BO_3_(3.1 g), MnSO_4_·4H_2_O (2.23 g), ZnSO_4_·7H_2_O (0.22 g), (NH_4_)_6_Mo_7_O_24_·4H_2_O (0.088 g), Co(NO_3_)_2_·6H_2_O (0.146 g), VOSO_4_·6H_2_O (0.054 g), Al_2_(SO_4_)_3_K_2_SO_4_·2H_2_O (0.474 g), NiSO_4_(NH_4_)_2_SO_4_·6H_2_O (0.198 g), Cd(NO_3_)_2_·4H_2_O (0.154 g), Cr(NO_3_)_3_·7H_2_O (0.037 g), Na_2_WO_4_·2H_2_O (0.033 g), KBr (0.119 g), KI (0.083 g), deionized water to 1 L], and 0.8 g/L agarose separately autoclaved and used for medium solidification) was used for cyanobacterial isolation. The water samples with low cyanobacterial abundance can be first concentrated and cultivated in an enriched medium, while those having high growth were diluted and subjected to a set of microbiological purification techniques. Density gradient centrifugation, serial dilutions, micromanipulation, repeated centrifuge-washing, and streak plating techniques are applied to attain axenic cyanobacterial cultures. Cyanobacterial plates were sealed with para-film, incubated at 25 ± 3 °C with continuous illumination with white fluorescence lamps at 1200 lx for 3–4 weeks, and examined daily under a light microscope.

### Set up of cyanobacterial culture and screening of the most producible isolates

Axenic cyanobacterial isolates were inoculated in 250 mL-volumetric flasks containing 100 mL sterile BG11 medium containing trace metal mix A5 [H_3_BO_3_(2.86 g), MnCl_2_.4H_2_O (1.81 g), ZnSO_4_.7H_2_O (0.222 g), Na_2_MoO_4_.2H_2_O (0.039 g), CuSO_4_.5H_2_O (0.079 g), Co(NO_3_)_2_.6H_2_O (49.4 mg)], supplemented with 1.7 g/L NaHCO_3_ and 1% NaCl. While nitrogen-fixing isolates were inoculated in BG0 medium without a nitrogen source. Cyanobacterial cultures were incubated for 30 days at 25 ± 3 °C with continuous illumination at 1200 lx using white fluorescence lamps. Using an Optical light microscope, the characterization of all species was approved based on classical algae keys. The morphological features were defined for each isolate, including cell shape, cell dimension, presence or absence of akinetes, heterocysts, calyptra, and sheath characters [19]. Then, cyanobacterial cultures were subjected to polysaccharide extraction and selection of the most potent isolate.

### Polysaccharide extraction procedures

#### Hot water extraction method for capsular polysaccharide

The crude polysaccharide was extracted using the hot water extraction method [20]. Three factors were optimized to obtain the maximum polysaccharide yield: the ratio of water to dried microalgae powder, extraction temperature, and extraction time. The cyanobacterial powder was extracted under a ratio of water to dried cyanobacterial powder (volume:dry weight, 30:1 mL:mg; extraction temperature (90 °C) and extraction time (4 h). The dried cyanobacteria powder mixtures were treated using an ultrasonic rod crasher at 1000 W for 10 min on ice to destroy cell walls. The mixtures were centrifuged at 7200 × g for 10 min and supernatants were collected, and the polysaccharide precipitated by the addition of absolute ethanol. The pellets were refluxed three times to remove any lipids with a mixture of acetone and chloroform (1:3, *v*/*v*). Residual proteins were removed using Sevage reagent (a 4:1 v/v mixture of chloroform and n-butanol). The protein precipitate was separated by centrifugation (1800 × g for 15 min) and discarded, and the supernatants were collected and dialyzed for 48 h and precipitated by the addition of ethanol. The pellets were dried using a lyophilizer for 12 h to obtain crude polysaccharide powder. The crude polysaccharide yield (%) was calculated as Yield % = Crude polysaccharide weight (mg)/dried microalgae powder (mg) × 100%.

#### Extraction of released polysaccharide using ice-cold ethanol

A volume of 1 L cyanobacterial culture broth was centrifuged at 1800 × g for 20 min at 4 °C. The supernatants were pressure-filtered through a rotary vacuum evaporator and the EPS was precipitated from the concentrated filtrate by the addition of three volumes of ice-cold absolute ethanol and the solution was kept at 4 °C overnight. Excess water was removed under a vacuum evaporator before lyophilization. The extracted EPS was lyophilized and stored at room temperature until chemical and physical analysis was performed. The crude polysaccharide yield (%) was calculated as mentioned above.

### Identification of the most potent cyanobacterial isolate

#### Morphological characterization and set up of growth curve

Axenic cyanobacterial culture identification was carried out using keys of Bergey’s Manual of Systematic Bacteriology [21]. The size, and morphology of the most potent cyanobacterial isolate were examined after fixation with 10% formaldehyde and coated with gold using a sputter coater (SPI-Module) using a scanning electron microscope (SEM) “JSM-5500 LV; JEOL, Ltd-Japan; by using high vacuum mode operating at 15 kV at the Central Laboratory, City of Scientific Research and Technological Applications, Alexandria, Egypt”. The effect of different NaCl concentrations (0, 10, and 20 g/L), light intensities (320, 640, 1280, 2000, and 2600 lx), and rpm (0, 50, 100, 120, 150, and 200) on the growth rate and polysaccharide productivity was studied and the optimal condition was selected for maximize the polysaccharide yield. To study the growth curve, the selected cyanobacterial isolate was grown in BG-11 broth medium supplemented with 10 g/L with continuous illumination (1280 lx using white fluorescence lamps) for 40 days at 25 ± 3 °C and 120 rpm. At each time interval (3 days), 2 mL of cyanobacterial culture was withdrawn and subjected to biomass determination and carbohydrate content using the phenol–sulfuric acid method.

#### Molecular identification of the most potent isolate and phylogenetic analysis

DNA was extracted from cyanobacterial isolate according to Saad et al. [22]. Briefly, 50 mL of cyanobacterial culture was harvested and washed three times with sterile distilled water and cyanobacterial pellets were well-grounded with 1 mL lysis buffer [0.2 M tris–HCl (pH, 7.4), 0.2 M EDTA, 4 M urea, and 20 mM NaCl)] and 100 µl proteinase K (20 mg/mL) and incubated 1 h at 60 °C in a water bath. Then, cyanobacterial DNAs were extracted using 1 mL extraction buffer (0.1 M tris–HCl (pH, 8), 3% CTAB, 20 mM EDTA, 1.4 M NaCl, 1% sarkosyl, and freshly added 1% mercaptoethanol). Then, the mixture was centrifuged for 20 min at 16,200 × g and the supernatant was added to an equal volume of phenol, chloroform, and isoamyl alcohol mixture (25:24:1 v/v) and centrifuged again for 15 min at 19,000 × g. The upper layer was added to an equal volume of cooled isopropanol for 30 min at −20 °C. After centrifugation, DNAase-free water was added to dissolve the DNA pellets and 1% agarose gel was used for cyanobacterial DNA genome electrophoresis. Polymerase chain reaction (PCR) was employed for amplification cyanobacterial 16S rRNA using specific primers (CYA106F; 5′ CGGACGGGTGAGTAACGCGTGA 3′, CYA781R (a); 5′ GACTACTGGGGTATCTAATCCCATT 3′, and CYA781R (b); 5′ GACTACAGGGGTATCTAATCCCTTT 3′). The PCR program was set as follows: initial denaturation at 95 °C for 3 min; 34 cycles of 94 °C for 1 min; annealing at 55 °C for 1 min; extension at 72°C for 1 min and a final extension step at 72 °C for 5 min. Then, an amplified product of the 16S rRNA gene was purified using a PCR clean-up column kit according to its manufacturing procedures. The sequences were submitted to the NCBI Genbank database, and the DNA sequence was aligned compared with other organism strains in the same species available in the Genbank database (http://www.ncbi.nlm.nih.gov). The neighbor-joining phylogenetic tree was created using version 11 of MEGA4 software [23].

### Purification of polysaccharide extracted from the most potent cyanobacterial isolate

The deproteinized polysaccharide solution (40 mg/20 mL) was subjected to dialysis against 20 mM phosphate buffer (pH, 7.2) in a cellulose membrane bag (molecular weight cut off 8000 Da) at 4 °C, and the precipitate formed during the dialysis treatment was removed by centrifugation at 1800 × g for 10 min. The resulting supernatant was applied on a DEAE-Sephacel (2.6 × 100 cm) column which had been equilibrated with 20 mM phosphate buffer (pH, 7.2). The column was washed with the same buffer, and then the bound polysaccharide was eluted using a gradient of NaCl from 0.0 to 1.0 M in 20 mM phosphate buffer (pH, 7.2) at a flow rate of 1.0 mL/min and fraction size 3.0 mL by AKTA prime plus fast protein liquid chromatography (FPLC). The carbohydrate content of the fractions was determined using the phenol sulfuric acid method [24], and the peptide content was determined using ultraviolet (UV) absorbance at 280 nm. The highly active fractions were collected, dialyzed, concentrated, and lyophilized for further analysis.

### Polysaccharide purity assessment using qualitative phytochemical analysis

To assess the polysaccharide purity, the presence of alkaloids, flavonoids, tannins, phlorotannins, terpenoids, steroids, saponins, phenols, and glycosides was tested [25]. These are rapid qualitative tests to detect the presence of impurities (other cyanobacterial compounds accompanied by cyanobacterial polysaccharides).

A qualitative method was employed to assess the presence of flavonoids. After dissolving 0.5 g of purified polysaccharide in 5 mL of 0.01 N NaOH, 5 mL of 0.01 N HCl was added. A yellow solution that becomes colorless indicates the presence of flavonoids. While the presence of alkaloids was determined by Mayer’s Test [26]. Briefly, 0.5 g of polysaccharide was dissolved in 10 mL of 0.1N HCl and filtered. The filtrate was then treated with a few drops of Meyer’s reagent, and 1 mL of 1% HCl was added to 3 mL of filtrate. The presence of alkaloids was indicated by a creamy white precipitate. The presence of tannins was detected using a ferric chloride test as follows. First, polysaccharide (0.5 g) was boiled in 10 mL of distilled water and then filtered. A few drops of 0.1% FeCl_3_ solution were added and the presence of tannins was determined by the appearance of a brownish-green or blue-black color. The presence of phlorotannins was detected by dissolving 0.5 g of polysaccharide in 5 mL distilled water and filtered. A red precipitate formed after boiling with a 2% HCl solution indicates the presence of phlorotannins [27].

The presence of terpenoids and steroids was assessed using a qualitative approach. Subsequently, 0.5 g of polysaccharide was mixed with 2 mL of chloroform. Then 3 mL of concentrated sulfuric acid was carefully added to form a layer. The presence of terpenoids was suggested by the interface’s reddish-brown color. The presence of saponin was assessed as follows, 5 mL of distilled water was used to dissolve 0.5 g of polysaccharide. Then, the solution was vigorously shaken, and steady persistent foam was seen. The foam was combined with three drops of olive oil and vigorously shaken, resulting in the production of a milky mass, indicating the presence of saponins [28]. Total phenolic compounds were tested using the protocol of González et al. [29]. A qualitative assay of the FeCl_3_ test was employed to assess the presence of phenolic compounds. About 0.1 g of polysaccharide was boiled with 1 mL of distilled water and filtered. The filtrate was then mixed with 2 mL of 1% FeCl_3_ solution. The presence of phenol was revealed by the formation of a blue-black color.

### Physicochemical and biochemical characteristics of the extracted polysaccharide

#### pH, color, and moisture content determination

The AOAC (Association of Official Analytical Chemists, 1884) technique was used to estimate the pH for purified cyanobacterial polysaccharides. The purified polysaccharide (1 g) was dissolved in 100 mL of dis water, and the pH was determined. In contrast to a white and dark lighting background, the color was evaluated by eye examination. The moisture of polysaccharides was determined by measuring the mass loss of purified polysaccharides after 24 h of heating at 105 ± 1 °C and expressed in % of EPS dry weight (w/w).

#### Carbohydrate content

According to Dubois et al. [24], the purified polysaccharide’s carbohydrate content was determined using the phenol sulphuric acid technique. One mL (0.1 g) of a cyanobacterial polysaccharide solution and 1 mL of a 5% phenol solution were mixed well and added to 5 mL of concentrated sulfuric acid. After the mixture was permitted to stand at 37 °C for 30 min, reading the absorbance at 490 nm was used to calculate carbohydrate concentration using mannose concentrations as a calibration curve.

#### Determination of uronic acid content

The carbazole test was used for uronic acid determination. In a 96-well plate, 20 µl of purified cyanobacterial EPS (5 mg/mL) or standard alginic acid (5 mg/mL) was serially diluted and then mixed with 80 µl of a 25 mM sodium tetra borate solution (prepared in sulfuric acid) solution and the mixture was heated for 10 min at 100 °C. After cooling at room temperature, 20 µl of 0.125% carbazole (prepared in absolute ethanol) was added, and the plate was heated again at 100 °C for 10 min. The plate was read at 550 nm after cooling to room temperature. Finally, the uronic acid content was expressed as % w/w of cyanobacterial EPS.

#### Determination of sulfate content

The turbidimetric method was used for the determination of sulfate content in cyanobacterial EPE. Cyanobacterial EPS (1 mg) was hydrolyzed using 0.25 mL of 0.5 M HCl and vortexed for 1 min. The mixture was incubated for 3 h at 105 ± 5 °C followed by centrifugation at 13,000 × g for 15 min after cooling at room temperature. Then, BaCl_2_-gelatin reagent was added to the gelatinous solution under stirring conditions. In a 96-well plate, 160 µl of acid hydrolyzed sample or standard solution (Na_2_SO_4_ at different concentrations) mixed with 40 µl of BaCl_2_-gelatine reagent and absorbance was detected at 405 nm after 20 min. the sulfate content was expressed as % w/w of cyanobacterial EPS using a standard calibration curve[30].

#### Composition analysis of the total protein, lipid, and ash

Briefly, polysaccharides (100 mg) were resolved with distilled water to 100 mL to make a polysaccharide solution of 1 mg/mL. Polysaccharide solution (1 mL) was put into a 10 mL tube, and a 5 mL solution of Coomassie brilliant blue G250 was added into the tube. After mixing, the optical absorbance was measured at 595 nm. Protein content expressed as %DW and bovine serum albumin were used for the calibration. According to AOAC guidelines, the total lipids were measured gravimetrically using the chloroform–methanol method. The ash percentage was detected according to the AOAC gravimetric approach as follows; about 1 g of the purified polysaccharide was burnt in a silica crucible for 24 h at 575 °C in a muffle furnace, weighed, and then its percentage was estimated after cooling.

### Characterization of cyanobacterial exopolysaccharide

#### Gas chromatographic-mass spectrometry (GC–MS) analysis

The chemical composition of tris (trimethylsilyl) methyl (TMS) derivatized polysaccharide was determined using a GC-TSQ mass spectrometer (Thermo Scientific, Austin, TX, USA) coupled with a TG-5MS direct capillary column (30 m × 0.25 mm × 0.25 m film thickness). The column oven temperature was initially kept at 60 °C, then increased by 6 °C/min to 250 °C with a 1-min hold, and then increased to 300 with a 30 °C/min increment. The temperature of the injector was kept constant at 270 °C. Helium was employed as a carrier gas at a constant flow rate of 1 mL/min. The solvent delay was 4 min, and a diluted sample of 1 µl was automatically injected using an Autosampler AS3000 linked with a GC in split mode. In full scan mode, EI mass spectra were collected at 70 eV ionization voltages spanning the m/z 50–650 range. The temperatures of the ion source and transfer line were set to 200 °C and 280 °C, respectively. The components were identified by comparing their mass spectra to the mass spectral databases NIST14 and WILEY 09.

#### Fourier transforms infrared (FTIR) spectroscopy analysis

Structural characteristics of the purified EPS were characterized by FTIR spectroscopy. The purified polysaccharide was crushed with pure potassium bromide, and the mixture was pressed into a small tablet [31]. The Shimadzu FTIR-8400 S spectrophotometer with a resolution of 1 cm^−1^ was used to measure the FTIR spectrum in the range of 4000 to 400 cm^−1^. The spectrum obtained can then be used to determine the functional groups present in the polysaccharide molecule.

#### Thermogravimetric analysis (TGA)

The purified cyanobacterial polysaccharide was subjected to TGA using a TGA-50H Thermo-gravimetric analyzer. The sample was scanned throughout a temperature range of 20 to 800 °C under a nitrogen atmosphere with a heating rate of 10 °C min^−1^ and a flow rate of 10 mL/min. The data are displayed as weight (%) vs temperature [32].

### Plackett–Burman design (PBD) for significant variable affection cyanobacterial polysaccharide production

Plackett–Burman (PBD) is a two-factorial design that is highly effective for screening the most important physicochemical parameters that are necessary for enhanced response in terms of their major effects [33]. Using a Plackett–Burman experimental design, the effects of fourteen nutritional and environmental parameters on cyanobacterial polysaccharide production were investigated. These factors included: A (NaHCO_3_, g/L), B (NaNO_3_, g/L), C (K_2_HPO_4_, g/L), D (MgSO_4_.7H_2_O, g/L), E (CaCl_2_, g/L), F (Citric acid, g/L), G (Ferric ammonium citrate, g/L), H (EDTA, g/L), J (Trace metal, mL), K (NaCl, g/L), L (Temperature, °C), M (pH), N (working volume, mL/100 mL volumetric flask), and O (Inoculum size, %, v/v) in addition to five dummy variables. A Plackett–Burman experimental design matrix with twenty runs was used to screen for relevant parameters influencing polysaccharide production during submerged fermentation. The parameters’ lower and higher levels are based on preliminary research. The Plackett–Burman design does not identify the mutual interactions between the processing parameters; rather, it serves as a method for screening and identifying significant parameters influencing the response. Therefore, the face-centered central composite design (FCCD) was employed to determine the levels of significant parameters and study the interaction effects among multiple significant parameters.

### Central composite design (CCD)

A Box-Wilson central composite design, also known as a central composite design (CCD), is widely used in response surface methods to develop a second-order polynomial for the response variables without employing a full factorial design of tests [34]. According to the findings of the Plackett–Burman experiment, CCD was used to analyze and optimize the levels of the parameters, as well as to study the interaction effects among the most significant independent variables affecting cyanobacterial polysaccharide synthesis. The most significant five variables (Working volume, EDTA, Inoculum size, CaCl_2_, NaCl) were selected and studied at five different levels which were two low (−2, −1), center (0), and two high (1, 2) levels. The zero levels (central values) chosen for the experiments were: Working volume (500 mL medium/1 L volumetric flask), EDTA (1.0 mg L^−1^), Inoculum size (7%), CaCl_2_ (0.036 g/L), NaCl (10 g/L). A total of 30 experiments were performed to optimize the levels and to study the interaction effects among the chosen factors on the cyanobacterial polysaccharide production by the selected isolate. Thirty runs were achieved in a 100 mL Erlenmeyer flask containing media prepared according to the design. After the media had been inoculated, they were incubated for 30 days at 1280 lx and 120 rpm.

### In vitro cytotoxic assay for purified polysaccharide from the selected isolate

The purified cyanobacterial polysaccharide was examined for their cytotoxic effect on the normal cell line (Vero cells) using MTT (3-(4,5-dimethylthiazol-2-yl)-2,5-diphenyltetrazolium bromide) assay [35]. Briefly, Vero cells (3 × 10^4^ cells/mL) were suspended in 10 mL of Dulbecco’s Modified Eagle Medium (DMEM) supplemented with 10% fetal bovine serum (FBS) and seeded in 96-well flat-bottomed plates. After incubation in a humidified 5% CO_2_ incubator for 24 h, the purified cyanobacterial polysaccharide was added to attached cells at concentrations of 100, 200, 500, 1000, and 2000 µg/mL. After incubation in a humidified 5% CO_2_ incubator for 3 days, the cells were washed three times with fresh medium, treated with 50 µl of 1 mg/mL MTT solution, and incubated at 37 °C for 3 h. Dimethyl sulfoxide (200 µl) was added to each well to dissolve the formed formazan crystals, and the optical density was measured at a wavelength of 570 nm. The percentage of viability compared to the untreated cells (control) was determined by the following equation: Cell viability (%) = (sample OD/control OD) × 100 and the calculations were performed at the required concentration and a 50% inhibition of viability (IC_50_) was determined [36].

### Antiviral activity of purified cyanobacterial polysaccharide

#### Antiviral assay using the cytopathic effect method

Vero cells (2 × 10^5^ cells/mL) were suspended in DMEM medium supplemented with 10% fetal bovine serum (FBS) and 1% l-glutamine and seeded in three 24-well microtiter plates for different three tests [22]. Plates were incubated in a humidified 5% CO_2_ incubator at 37 °C till cell monolayer became 100% confluent and subjected to three different mechanisms as follows,

#### Neutralizing effect of the purified cyanobacterial polysaccharides

The purified polysaccharide was incubated with 100 µl TCID_50_ (50% tissue culture infectious dose) of different viruses (30 PFU/100 µl) at final concentrations of 5, 10, and 20 µg/mL for 2 h at 4 °C. After incubation, different mixtures were added to monolayer cells and incubated in a humidified 5% CO_2_ incubator at 37 °C for 2–4 h. Then, mixtures were discarded, and cells were washed three times with fresh DMEM medium. Finally, the overlaying medium was carefully added to the infected monolayer cells and incubated in a humidified 5% CO_2_ incubator at 37 °C for three days before plaque detection.

#### Protection activity of the purified polysaccharides

The purified cyanobacterial polysaccharide was suspended in DMEM medium and added to cells at final concentrations of 5, 10, and 20 µg/mL and incubated at 37 °C for 2 h. After a 2 h incubation period, Vero cells were washed three times with fresh DMEM medium, infected with 100 µl TCID_50_ of different viruses, and incubated at 37 °C for 2 h. After that, cells were washed three times with fresh DMEM medium followed by the addition of 1 mL of overlaying medium for each well, and incubated in a humidified 5% CO_2_ incubator at 37 °C for 3 days. Finally, formed plaques were detected and enumerated as previously described.

#### Effect of the purified polysaccharide against intracellular replication

Vero cells were infected with 1 mL TCID_50_ of different viruses and incubated at 37 °C for 2 h. The purified polysaccharide was mixed with 1 mL of overlaying medium to be at final concentrations of 5, 10, and 20 µg/mL and carefully overlaid on the top of monolayer infected cells after 2 h. The number of plaques was detected after incubation in a humidified 5% CO_2_ incubator at 37 °C for 3 days. Acyclovir was used as a positive control at the concentration of 10 μg/mL. Finally, data were expressed as 50% inhibitory concentration (EC_50_), which was defined as the drug concentration that prevented 50% of viral plaque formation when compared to untreated control cells. The percentage of viral inhibition was estimated using the following equation: [(number of plaques in positive control − number of plaques in tested sample)/ number of plaques in positive control] × 100.

#### Antiviral activity of purified cyanobacterial polysaccharide using the MTT method

Vero cells were conducted on three different trajectories mentioned above. The first trajectory examined the neutralizing effect of purified cyanobacterial polysaccharides. Briefly, the purified polysaccharide was incubated with 100 µl TCID_50_ of different viruses (30 PFU/100 µl) at final concentrations of 5, 10, and 20 µg/mL. After incubation at 4 °C for 2 h, these mixtures were added to monolayer cells and incubated for 2 h in a humidified 5% CO_2_ incubator at 37 °C. Then, suspensions were discarded, and Vero cells were washed three times with fresh DMEM medium before incubation for three days in DMEM medium supplemented with 10% fetal bovine serum (FBS) and 1% l-glutamine. The second trajectory examined the protecting ability of cyanobacterial polysaccharide as follows; the purified polysaccharide was added to cells at final concentrations of 5, 10, and 20 µg/mL and incubated at 37 °C for 2 h. After incubation, Vero cells were washed three times with fresh DMEM medium, and then cells were infected with 100 µl TCID_50_ of different viruses and incubated at 37 °C for 2 h. After that, Vero cells were washed three times with fresh DMEM medium and incubated for three days in DMEM medium supplemented with 10% FBS. Finally, the third trajectory examined the inhibitory effect of purified polysaccharide for different viral replication in Vero cells as follows; Vero cells were infected with 100 µl TCID_50_ of different viruses and incubated at 37 °C for 2 h. Then, Vero cells were washed three times with fresh medium and the purified polysaccharide was added at final concentrations of 5, 10, and 20 µg/mL to infected cells after 2, 4, 6, and 24 h and then incubated in DMEM medium supplemented with 10% FBS for three days in humidified 5% CO_2_ incubator at 37 °C. Finally, the percentage of cell viability and EC_50_ was measured as previously described.

### Statistical analysis

Data were represented as mean ± SD using graph pad prism (version 9). Data significance was analyzed using SPSS (Statistical Package for the Social Sciences) Statistics software (version 29.0.2.0). The Windows edition of design-expert software (version 13, Stat-Ease, Minneapolis, USA) was used for the experimental design and statistical analysis (https://www.statease.com/sostware/design-expert/).

## Result and discussion

### Screening and selection of the most potent EPS-producing cyanobacteria

**Fig. 1:**
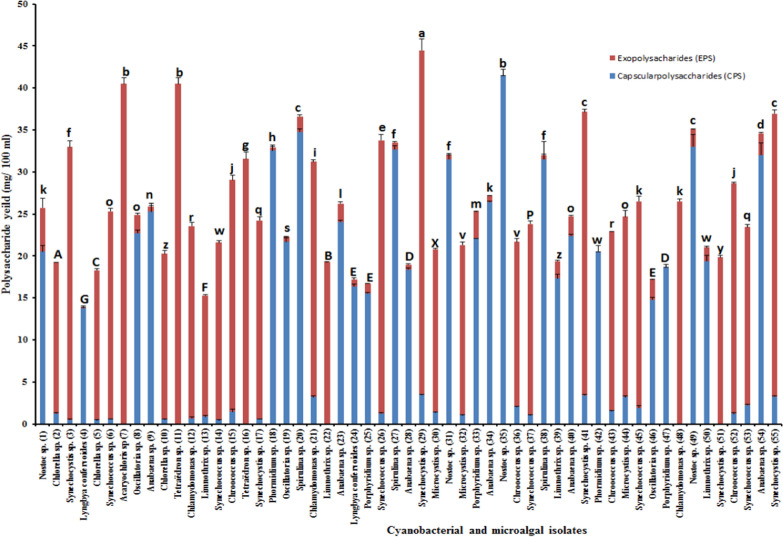
Screening for the most potent polysaccharide-producing microalgae isolates culturing on BG11 or BG0 medium, and incubated in a rotary shaker for 30 days at 25 ± 3 °C with continuous illumination at 1200 lx. Different letters exhibit significant differences, as determined by Duncan’s multiple range tests (*P* ≤ 0.05) conducted using SPSS Statistics 29.0.2.0

Fifty-five (15 microalgae and 40-cyanobacteria) strains were successfully isolated and conducted to our survey based on the cover of a maximum of phylogenetic diversity, target classes to produce EPS, selected species to study the diversity of compositions within the same genus and chosen strains with a promising phenotype as both biofilm and gel formations or slimy appearance during the cultures. The morphological criteria outlined in Bergey’s Manual of Systematic Bacteriology [21] were employed to initially identify axenic microalgal and cyanobacterial isolates. In instances where the genus and species names are unknown, brief morphological descriptions of the strains can be found in the Roscoff Culture Collection (http://roscoff-culture-collection.org/). To identify positive strains, two conditions were considered: growing under the chosen conditions [Microalgal and cyanobacterial cell suspension (2 × 10^7^ cell/mL) were prepared, inoculated in 100 mL modified BG11 or BG0 (for nitrogen-fixing isolates), and incubated in a rotary shaker for 30 days at 25 ± 3 °C with continuous illumination at 1200 lx] and producing a minimum of 70 mg L^−1^ of true EPS after 30 days of incubation. Our survey showed that the polysaccharide-producing isolates were classified into three groups: capsular polysaccharide producer, extracellular polysaccharide producer, and fractional polysaccharide producer. *Acaryochloris* sp. (isolate number 7) was selected for subsequent investigation based on mentioned criteria for further study as it produced a significant amount of extracellular polysaccharide; 405.7 ± 0.01 mg L^−1^ (Fig. 1). Recently, there has been a growing interest in marine sulfated polysaccharides (MSPs) because of their promising antiviral properties. *Acaryochloris* sp. is the only marine microalgae strain successfully isolated in our survey. Its exopolysaccharide was selected for antiviral investigation as the previous studies reported that marine microalgae and macroalgae produced a significant amount of sulfated exopolysaccharide (SPs) with a wide range of antiviral activity against several viruses, including HSV-1 and HSV-2 [37, 38], VSV, pseudorabies virus (PRV) [38], influenza virus [39], encephalomyocarditis virus (EMCV) [40], rotaviruses, infectious hematopoietic necrosis virus (IHNV), infectious pancreatic necrosis virus (IPNV) [41], and African swine fever virus (ASFV) [42].

### Identification of the most potent EPS-producing isolate

#### Cyanobacterial growth kinetic and EPS production

**Fig. 2:**
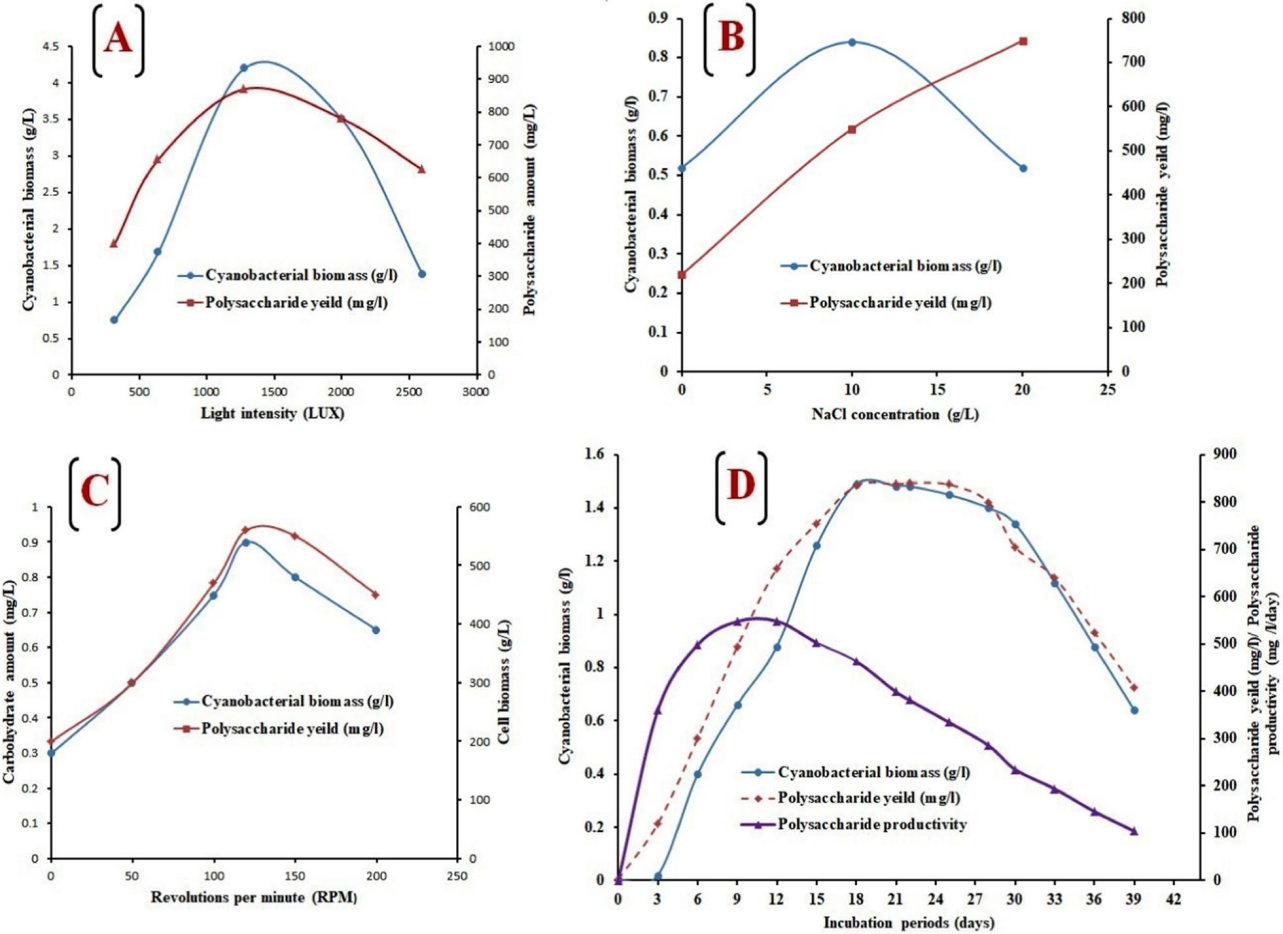
The effect of different parameters on growth and polysaccharide production from *Acaryochloris* sp., including light intensities (**A**), NaCl concentrations (**B**), and revolutions per minute (RPM), where (**D**) displaying the growth pattern, polysaccharide amount, and productivity at 25 ± 3 °C for 40 days

The optimal growth curve of *Acaryochloris* sp. on a lab scale was conducted using BG 11 medium supplemented with NaCl (10 g/L) with continuous illumination at 1280 lx, and 120 rpm (optimal for growth and EPS production (Fig. 2A–C) at 25 ± 3 °C for 40 days (Fig. 2D). Under this condition, *Acaryochloris* sp. displays a typical growth curve with a clear exponential phase observed from day 3 to day 18 of culture time and maximum cyanobacterial biomass of 1.48 g/L. This result is far higher than that obtained in the case of *Chlorella vulgaris* (1.1 g/L) and *Dunalilella salina* (0.997 g/L), which are considered the main chlorophytes used in industrial production [43, 44]. Total EPS production by *Acaryochloris* sp. gradually increased during the exponential growth phase and maximized production was detected during the stationary phase (Fig. 2D). The maximum yield was 810 ± 0.01 mg L^−1^ of EPSs, which occurred at 22nd of a cyanobacterial growth phase. This amount was higher than that of EPSs from *Anabaena cylindrical* 10 C (2.36 mg L^−1^), *Arthrospira platensis* (11.76 mg L^−1^ day^−1^) [45], and *Oscillatoria formosa* (9.88 mg L^−1^ day^−1^) [46], and lower than those recorded for optimized cyanobacterial laboratory cultures conditions for example, *Anabaena* sp. *ATCC 33047* (1100 mg L^−1^) [47]. Moreover, as shown in (Fig. 2D), there is a strong correlation linking the kinetics of cellular growth and EPS production, with a maximum daily productivity of up to 548.33 mg L^−1^ day^−1^ for both biomass and EPSs detected at 12 days of the growth. This is the first documentary reported on exopolysaccharide production from *Acaryochloris* sp. isolated from ponds. The different physicochemical factors (suboptimal temperature, limited nutrients, and osmotic stress) that restrict cellular activities may enhance microbial exopolysaccharide production [48–50]. Our observations confirm that our isolate; *Acaryochloris* sp. with a slow growth rate can survive in high salinity conditions by producing exopolysaccharides in the vicinity of the outer cell boundary. Under salinity stress, bacterial cells invest in increasing exopolysaccharide production and slow down the growth rate to save cellular energy for survival rather than replication. An enhanced exopolysaccharide layer can protect the cells from dehydration by creating a microenvironment surrounding the cell wall that buffers the osmotic disequilibrium across the cell membrane provides a repository for water and slows down the ion influx under hyper saline conditions [51, 52].

#### Morphological and molecular identification of the most potent isolate

**Fig. 3:**
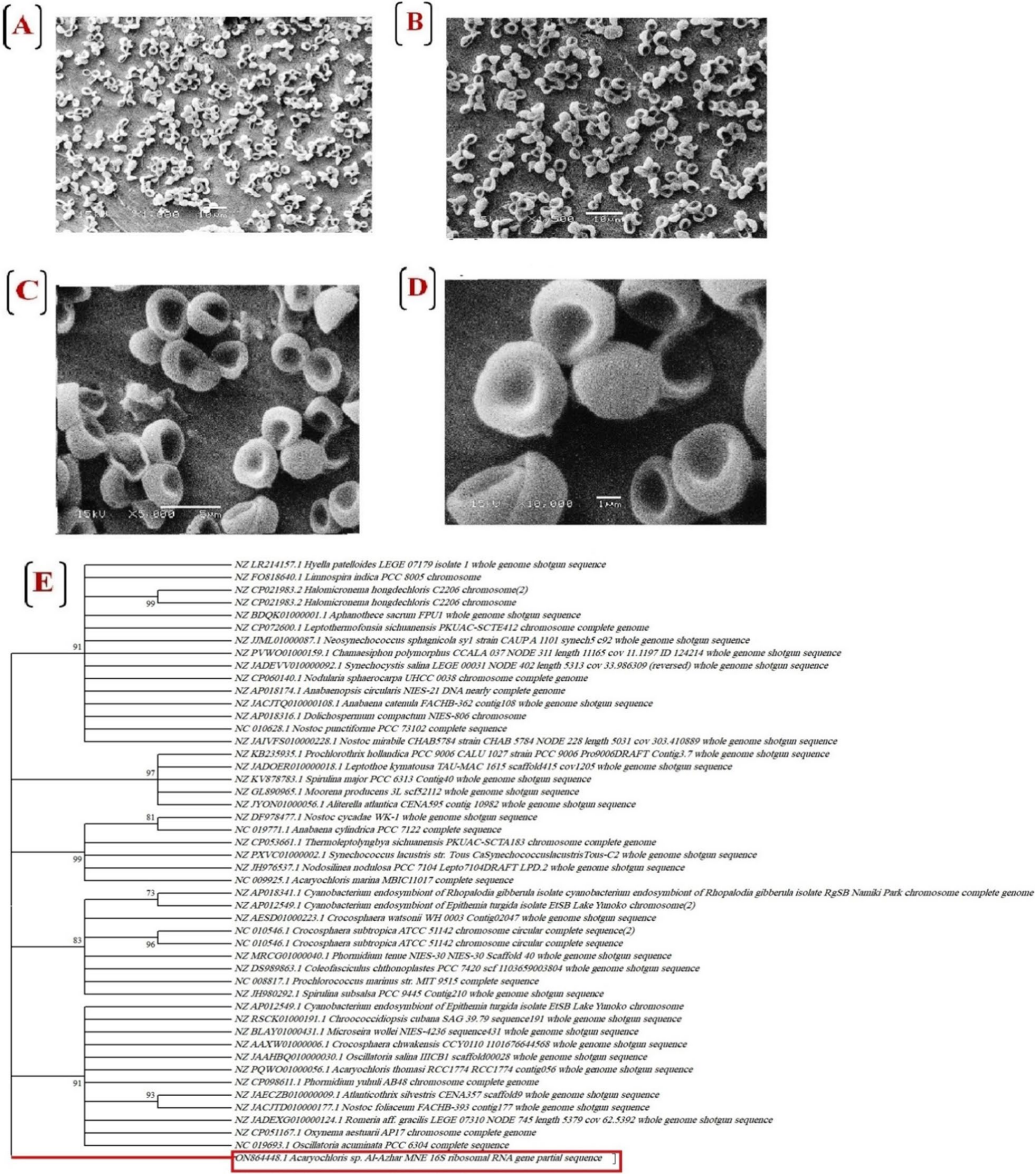
**A–D** Scanning electron microscopy showing cell morphology of *Acaryochloris* sp. isolate at different magnification power. **E** the phylogenetic tree of *A. Al-Azhar MNE ON864448.1*., and the evolutionary distances were calculated using the Kimura 2 model using MEGA version 11

The axenic cyanobacterial culture of our isolate was morphologically identified under a light microscope. Using a light microscope at 100× magnification power, our cyanobacterial isolate appears as non-motile, blue-green, coccoid bacteria. Cyanobacterial cells tend to assemble into clumps. Based on keys of Bergey’s Manual of Systematic Bacteriology [21], and Desikachary [53], our isolate was classified into *a genera; Acaryochloris*, family; *Acaryochloridaceae*, order; *Chroococcales*, class; *Cyanophyceae*, division; *Cyanophyta* of Bacterial Domain. Scanning electron micrograph (Fig. 3A–D) shows that *Acaryochloris* cells are spherical with a cell diameter of ~ 2 µm. While the 16S rRNA gene sequence of our cyanobacterial isolate; *Acaryochloris* sp. was compared with sequences from a wide range of other cyanobacteria representative of the large genetic diversity existing within this ancient group. Moreover, the basic local alignment search tool (BLAST) revealed that, the 16S rRNA sequence of our cyanobacterial isolate shares 96% identity, 91% query cover, and 2e-135 E-value with *Acaryochloris marina* MBIC11017 and 90% identity, 89% query cover, and 4e-101 E-value with *Acaryochloris thomasi* RCC1774. All three tested phylogenetic methods (Maximum Likelihood/Neighbor-Joining/Bayesian inference) consistently placed our cyanobacterial isolate in a new taxon (Fig. 3E). Depending on the phylogenetic analysis results within the morphological characteristics, our cyanobacterial isolate was classified as a new isolate*, Acaryochloris Al-Azhar MNE *and deposited in the Gene bank with accession number, *ON864448.1*.

### Purification and purity examination of cyanobacterial exopolysaccharide

**Fig. 4:**
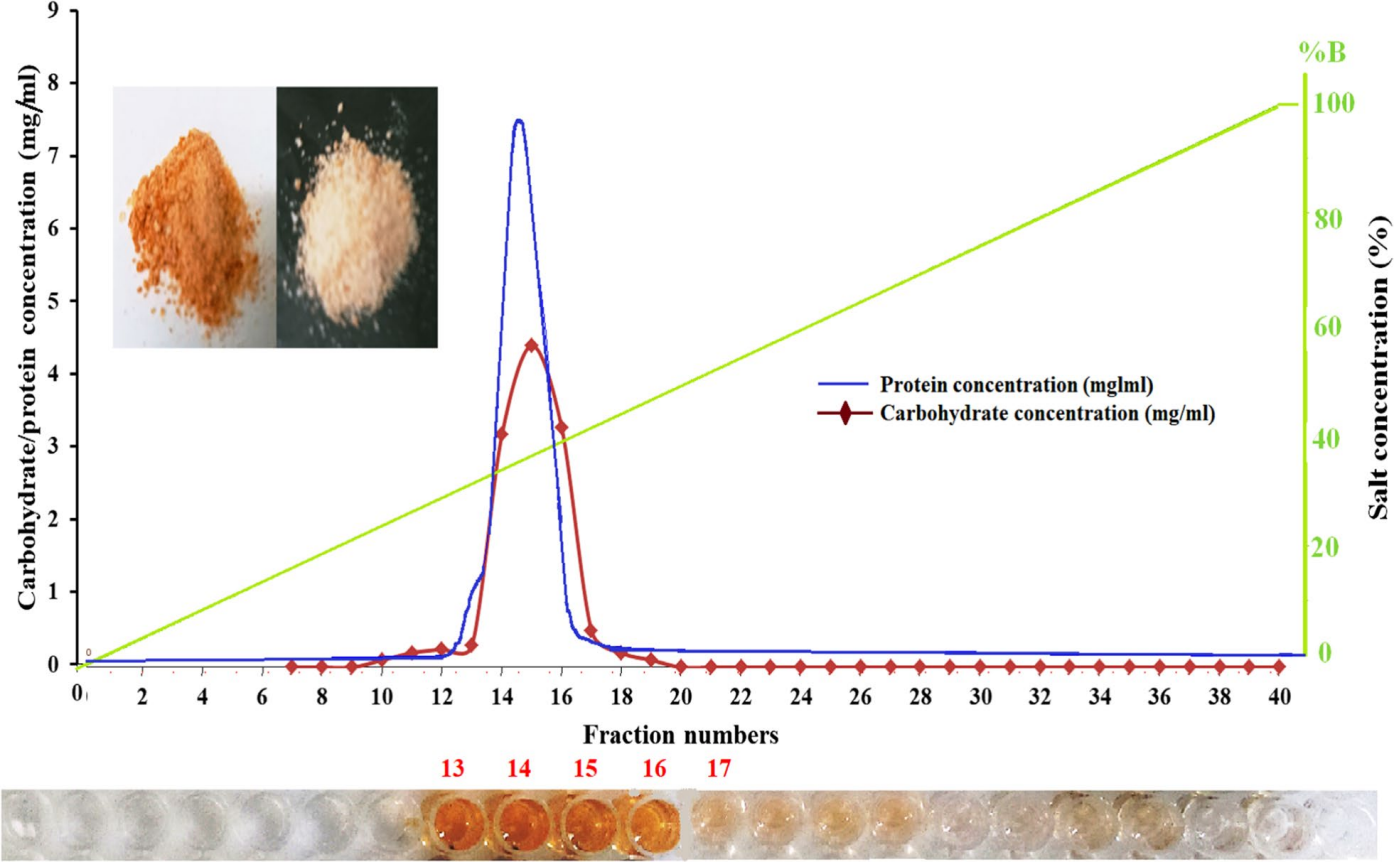
Chromatographic purification of *A. Al-Azhar MNE ON864448.1* EPS. The crude extract was applied to the DEAE-52 cellulose column equilibrated with phosphate buffer (pH, 7.2). The column was washed with phosphate buffer (pH, 7.2) and eluted with gradients of NaCl using AKTAprime plus a fast-performance liquid chromatography (FPLC) protein separator system

After polysaccharide extraction, ethanol precipitation, defatting, deproteinization, and lyophilization, the crude polysaccharide (40 mg/20 mL) was subjected to ion exchange chromatography purification. The lyophilized polysaccharide was dissolved in distilled water and then applied to DEAE-52 cellulose ion exchange chromatography. The column was eluted with 20 mM phosphate buffer (pH, 7.2) and different stepwise gradients of NaCl solutions at a flow rate of 1.0 mL/min. The elution profile of EPS extracted from *A. Al-Azhar MNE ON864448.1* displays only one single symmetrical peak (Fig. 4). The phenol–sulfuric acid assay showed that fractions 13, 14, 15, and 16 recorded the highest polysaccharide content with a total amount of 33.5 mg/12 mL. The ion exchange column yielded 83.75% of purified cyanobacterial EPS indicating the effectiveness of the chromatography technique for cyanobacterial EPS purification especially after the deproteinization step. According to phytochemical tests for polysaccharide purity examination, a polysaccharide derived from column chromatography was free of all phytochemical components, including terpenoids, flavonoids, phlorotannins, tannins, alkaloids, saponins, glycosides, phenols, and steroids, any accompanying cyanobacterial metabolites may be hindered the extracted polysaccharide to be further applied in different sectors. Our cyanobacterial isolate exhibits a remarkably high yield of cyanobacterial EPS, which is also characterized by its high purity. This achievement may pave the way for its potential application in the field of industry. The production of bacterial EPS at an industrial scale offers significant advantages over the production of biopolymers from other natural sources: (1) they are typically actively secreted by the cells, making them easy to extract and purify, thus reducing production costs; (2) the strains used usually have fast growth rates, resulting in accelerated production processes; (3) they are not subject to the strict environmental regulations applied to animal or plant-based polymer production, such as ethical concerns, animal protection, or deforestation prevention; and (4) in certain cases, the polymers and/or the producing strain can be easily modified or engineered to achieve desired properties and/or improved performance. Furthermore, the production of EPS by cyanobacteria offers two additional advantages in comparison to the production of bacterial EPS that is currently being commercially exploited: cyanobacteria have simple nutritional requirements and the cyanobacterial EPS are more complex and versatile, making them suitable for various applications in high-value market niches such as cosmetic, pharmaceutical, and biomedicine [54]. However, despite the abundance of available data, the practical implementation of cultivating, extracting, and purifying this polymer remains unrealized. This is necessary to effectively control the essential criteria for medical applications, including purity, stability, and safety. Moreover, a comprehensive understanding of the cyanobacterial EPS biosynthetic pathways is still required to optimize the customized production of specific EPS and to modify their structure and composition to suit specific applications.

### Characterization of cyanobacterial exopolysaccharide

#### Physicochemical and biochemical characteristics

The cyanobacterial exopolysaccharide is part of a complex network of extra polymeric substances, which comprise other compounds; nucleic acids, lipids proteins, and secondary metabolites [55]. The physicochemical property of *A. Al-Azhar MNE ON864448.1* EPS was displayed in Table 1. The color of the extracted polysaccharide after purification was yellow-white and the polysaccharide solution (1 g/mL) recorded a pH value of 8.5. While the moisture content of purified cyanobacterial polysaccharide equaled 10.2 ± 0.5%. The biochemical tests displayed that, the EPS purified from *A. Al-Azhar MNE ON864448.1* shows positive observation with Molisch’s and iodine tests indicating the presence of carbohydrates while phenol–sulfuric acid test accounts for 85 ± 2.5% of this EPS as a carbohydrate. BaCl_2_-gelatin assay displayed that sulfate constitutes 1.7 ± 0.5% of *A. Al-Azhar MNE ON864448.1* EPS. Uronic acids have an exclusive occurrence in the cyanobacterial polysaccharide, being identified with a frequency of one or two units. Using standard alginic acid, the amount of uronic acid present in the purified cyanobacterial polysaccharide was quantitatively determined. We found that uronic acid constitutes 9.5 ± 2.1% of *A. Al-Azhar MNE ON864448.1* polysaccharide. More than 13 different monosaccharides have been reported to be a polymeric unit for cyanobacterial polysaccharides which grouped into pentoses (arabinose ribose, and xylose), deoxyhexoses (2-O-methyl-L-rhamnose, 3-O-methyl-L-rhamnose, rhamnose, and fucose), hexoses (fructose, glucose, mannose, and galactose) and acidic hexoses (galacturonic and glucuronic acid) [56]. The biochemical tests showed negligible contents of other concomitants like ash, proteins, and phenols.

**Table 1:** The physicochemical and biochemical properties for EPS purified from *A. Al-Azhar* MNE *ON864448.1*

Physiochemical properties		Biochemical properties	
pH	8.5	Carbohydrate content	85 ± 2.5%
		Total protein content	0.070 ± 0.1%
Color	Yellow-white	Sulfate content	1.7 ± 0.5%
		Phenol content	0.7 ± 0.32%
Moisture content	10.2 ± 0.5%	Lipid content	0
		Ash content	0.65 ± 0.5%

#### Fourier-transform infrared (FT-IR) spectroscopy

**Fig. 5:**
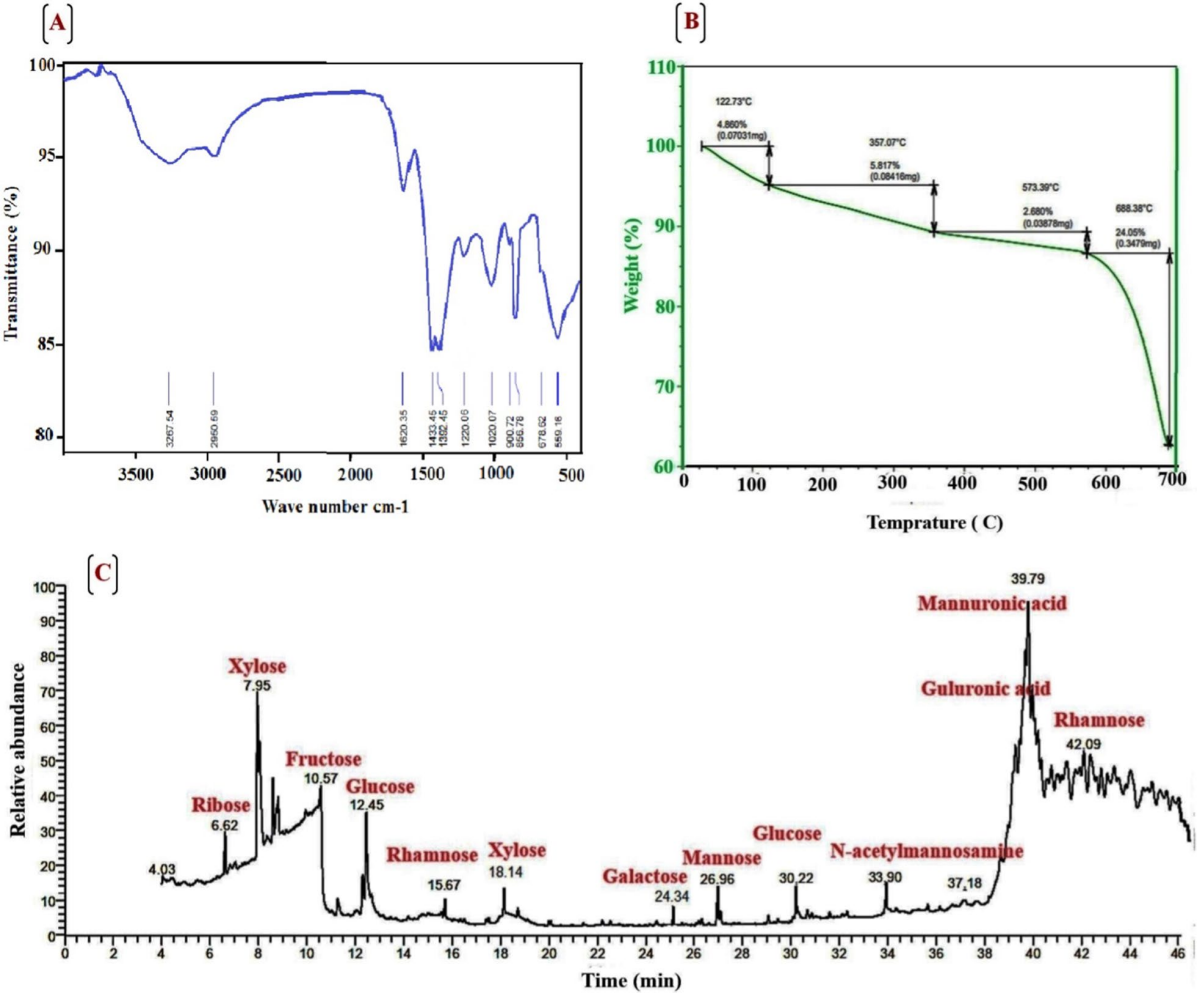
Displaying **A** Fourier-transforms infrared (FT-IR) spectroscopy, **B** TGA thermogram, and **C** GC/MS chromatogram for TMS-derivatized sugars of purified *A. Al-Azhar MNE ON864448.1* EPS

Fourier-transform infrared (FT-IR) spectra provided a useful guide for polysaccharide identification through well-divided absorption bands of characteristic groups [57]. The FTIR spectrum of the EPS produced by *A. Al-Azhar MNE ON864448.1* revealed several characteristic absorption bands (Table 2 and Fig. 5A). A strong abroad band was observed in the region of 3267.54 cm^−1^ and weak band at 2950.59 cm^−1^, which could be attributed to the stretching vibration of O–H and C–H groups, respectively, typical of hydroxyl and alkyl functionality of carbohydrates [58]. The sharp vibration at 1620.35 cm^−1^ corresponds to asymmetric stretching vibration belonging to (C=O) of the carboxylate ion (COO–). The region between deformational vibrations 1500–1200 cm^−1^ is called the local symmetry region. The strong symmetrical vibrations at 1433.45 and 1392.45 cm^−1^ are corresponding to (–CH2) scissoring and (–OH) bending vibrations, respectively. The vibrational band around 1220.06 is related to the asymmetric vibration of the sulfate group (S=O) indicating that EPS extracted from *A. Al-Azhar MNE ON864448.1* structurally involves sulfate groups. The region of frequencies between 800 and 1200 cm^−1^ is called the fingerprint region. The sharp band detected at 1020.07 cm^−1^ corresponded to the vibration of the C–O group of uronic acid [59]. The characteristic vibration at 800–900 cm^−1^ suggested polysaccharide extracted from *A. Al-Azhar MNE ON864448.1* has α- and β-configuration or two different glycosidic linkages. The region with wavenumbers at 856.78 cm^−1^ belongs to an additional sulfate absorption band (C–O–S) [60]. The vibrational region below 800 cm^−1^ is called a skeletal region. The absorption band at 559.16 cm^−1^ is corresponding to the symmetrical vibration of the sulfate groups (O–S–O). Overall, these results indicated that *A. Al-Azhar MNE ON864448.1* EPS possessed typical absorption bands of polysaccharides [59].

**Table 2:** FTIR frequencies and functional groups for *A. Al-Azhar MNE ON864448.1* EPS

Wave number (cm^−1^)	Vibration shape	Assignment groups
3267.54	Broad band	O–H
2950.59	Weak band	C–H
1620	Weak band	Asymmetric vibration belongs to (C=O) of the carboxylate ion (COO–)
1433.45 and 1392.45	Scissoring strong band	(–CH2) scissoring and (–OH) bending vibrations, respectively
1220.06	Weak band	The asymmetric stretching of the sulfate group (S=O)
1020.07	Medium band	C-O group of uronic acid
856.78	Strong sharp band	Secondary axial sulfate (C–O–S)
559.16	Strong band	The symmetrical vibration of the sulfate group (O–S–O)

#### Thermogravimetric analysis (TGA)

Figure 5B displays the TGA thermogram of *A. Al-Azhar MNE ON864448.1* EPS. This cyanobacterial polysaccharide exhibits four regular transition stages. In the first stage (**Ι**), the TGA thermogram shows a slight decrease in weight (0.0703 mg corresponding to 4.86% of EPS) from room temperature to 122.73 °C, which was attributed to dehydration or losing the physically bound water molecules being attached to the carboxyl groups [61]. While the second stage (**II**) which corresponds to elevating temperature from 122.73 to 357.07 °C displays a weight loss of 0.08416 mg equivalent to 5.817% of EPS. The weight loss during this stage is primarily attributed to the breakdown of thermally unstable functional groups. Thermal characteristics of step (**III**) were detected when the temperature elevated above 357.07 °C with a weight loss of 2.68% (corresponding to 0.03878 mg) until the temperature reached 573.39 °C. The weight loss at this stage is explained by polysaccharide de-polymerization and de-composition (thermal breakdown) which resulted in the breakdown of C–C and C–O bonds in pyranose ring structures and generation of H_2_O, CO, and CO_2_. The last stage (**IV**) was reached when the temperature elevated to 688.38 °C, and chars formed. The weight loss of *A. Al-Azhar MNE ON864448.1* EPS at this stage equals 0.3479 mg corresponding to 24.05% of EPS. It is concluded that polysaccharide extracted from *A. Al-Azhar MNE ON864448.1* is thermostable until 357.07 °C. This cyanobacterial polysaccharide’s remarkable thermostability may be related to the presence of uronic acids, which prevents complete polymer breakdown. The presence of galacturonic acid and xylose was shown to have a role in the development of pyrolysis according to [62].

#### Gas chromatographic–mass spectrometry (GC–MS) analysis

GC/MS analysis for TMS-derivatized sugars showed that *A. Al-Azhar MNE ON864448.1* EPS is composed of at least ten different types of monosaccharides (Fig. 5C). This finding illustrated a high proportion of two different types of uronic acid (guluronic acid; 4.98% and mannuronic acid; 5.51%) providing a high affinity for positively charged molecules (*e.g.*, metal cations) and the presence of pentoses (xylose; 8.85% and ribose; 2.72%) usually absent in other EPSs of prokaryotic origin. The GC chromatogram for TMS-derivatized sugars exhibited absorbance about hexose sugars, specifically fructose (4.29%), glucose (6.35%), galactose (2.64%), and mannose (3.09%). This observation aligns with the findings reported for multiple strains, including *Nostoc* sp. and *O. Formosa* [63]. Other notable characteristics of the *A. Al-Azhar MNE ON864448.1* EPS were the presence of sugar derivatives (N-acetylmannosamine; 2.67%), and the occurrence of deoxyhexoses (rhamnose; 7.97%), enhancing the hydrophobicity of *A. Al-Azhar MNE ON864448.1* EPS. Several publications concerned with cyanobacterial EPS reported that EPS extracted from cyanobacteria are heteropolymers, with more than 13 distinct monosaccharide monomers, which is more than those produced by other bacteria (which include 4 different monomers) [56]. Monosaccharide building blocks usually identified in cyanobacterial EPS are pentoses (arabinose, ribose, and xylose), hexoses (glucose, mannose, galactose, and fructose), acidic hexoses (galacturonic and glucuronic acids), the amino sugars (N-acetylglucosamine, glucosamine, N-acetylgalactosamine, and galactosamine) and the deoxyhexoses (rhamnose and fucose) [56]. The presence of uronic acid and sulfate groups in the cyanobacterial EPS is responsible for their anionic charge, which makes them valuable for use in a variety of life sectors [64]. The amphiphilic property of cyanobacterial EPS is attributed to the presence of both the hydrophilic part including uronic acids, ketal-linked pyruvyl groups, and sulfated sugars, and the hydrophobic part including deoxysugars (e.g., fucose and rhamnose), ester-linked acetyl groups, and polypeptide moiety [56, 65, 66]. These hydrophobic and hydrophilic groups also play an important role in the emulsifying properties of cyanobacterial polysaccharides [56, 67]. Overall, the characteristics are mainly responsible for polymer functionalization, making them unique molecules with diverse applications in biotechnological and medicinal fields.

### Application of Plackett–Burman design to assess the significance and effect of process variables on cyanobacterial polysaccharide production

**Fig. 6:**
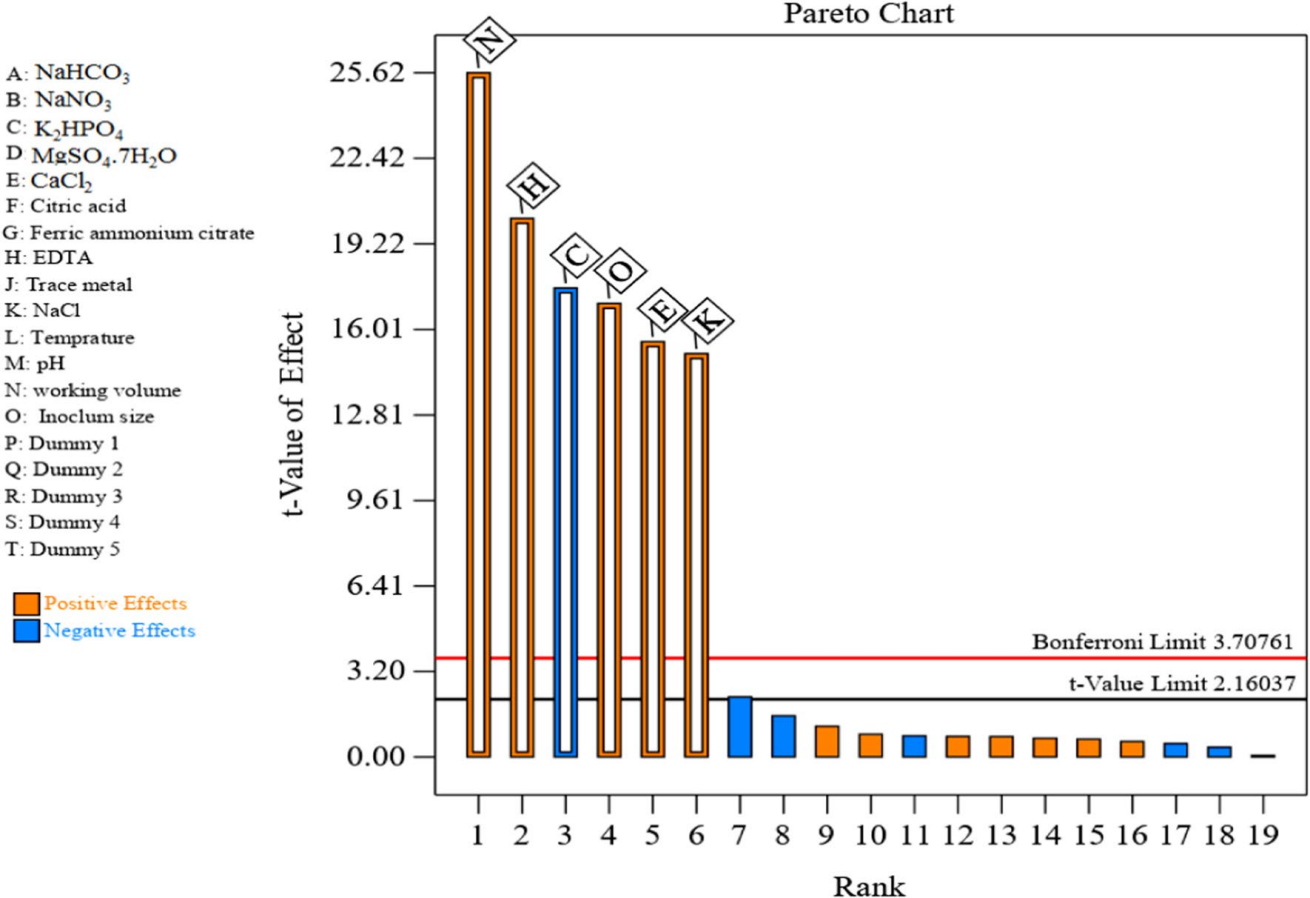
The Pareto chart assesses the effects of independent variables on EPS production from *A. Al-Azhar MNE ON864448.1* (the blue color symbolizes the negative effect and the orange color symbolizes the positive effect)

Initially, Plackett–Burman statistical design was employed to evaluate the effects of fourteen nutritional and environmental variables, including A (NaHCO_3_, g/L), B (NaNO_3_, g/L), C (K_2_HPO_4_, g/L), D (MgSO_4_.7H_2_O, g/L), E (CaCl_2_, g/L), F (Citric acid, g/L), G (Ferric ammonium citrate, g/L), H (EDTA, g/L), J (Trace metal, mL), K (NaCl, g/L), L (Temperature, °C), M (pH), N (working volume, mL/100 mL volumetric flask), and O (Inoculum size, %, v/v) on cyanobacterial polysaccharide production by the selected strain. Secondly, a face-centered central composite design was employed to optimize significant variables and investigate their interactions with cyanobacterial polysaccharide synthesis. In comparison to other growth medium design strategies, the Plackett–Burman design is a simple and quick method for screening a large number of variables in one experiment to evaluate the significant variables affecting the cultural requirements and polysaccharide production in submerged fermentation [68, 69]. It was carried out by performing 20 runs to identify the key parameters that increase cyanobacterial polysaccharide production by the selected strain. Additional file 1: Table S1 illustrates the experimental design and the fourteen independent parameters as well as their levels used in the experimental design. Results of different tests show a variation in findings from 0.22 to 0.77 mg/mL. This change reflects the importance of medium optimization to achieve high polysaccharide production. The minimum polysaccharide production (0.22 mg/mL) was observed in the third run, while the maximum polysaccharide production (0.77 mg/mL) was achieved in the eighth run. To determine the correlations between the independent variables and polysaccharide production, a multiple-regression mathematical model was used. Statistical analysis was achieved and summarized in Table 3 and Additional file 1: Table S3. Table 3 and Fig. 6 show the estimated effect of the investigated variables on polysaccharide synthesis. The main effect permits the evaluation of each factor’s influence on polysaccharide synthesis. Both large positive or negative effects demonstrate that a variable has a strong effect on production, whereas the factor has little effect or is considered ineffective with a value near zero. From the main effect results, we can find that five parameters named working volume (N), EDTA (H), inoculum size (O), CaCl_2_ (E), and NaCl (K) have a positive influence on the polysaccharide synthesis and one parameter namely K_2_HPO_4_ (C) negatively affect the polysaccharide synthesis while other parameters slightly affected polysaccharide synthesis. The contribution percentage of each parameter is presented in Table 3; working volume (N), EDTA (H), K_2_HPO_4_ (C), inoculum size (O), CaCl_2_ (E), and NaCl (K) are the most contributing components with 30.46, 18.9, 14.3, 13.45, 11.29, and 10.64%; respectively. Parameters with positive impacts on polysaccharide production have been used at a high level, while the parameters that have a negative effect are kept at a low level for further optimization by face-centered central composite design. The Pareto chart offers a simple approach to view the results of the Plackett–Burman design by illustrating the significant order of the variables influencing cyanobacterial polysaccharide synthesis (Fig. 6). It displays the impacts’ absolute values and draws a reference line on the chart. Any influence that extends above this reference line has the potential to be significant. Using Design Expert version 13.0, the Pareto graphic reproduces the relation between variable effects (t-value) vs. ranks. Among the 14 assigned variables, working volume (N) was the most significant variable affecting cyanobacterial polysaccharide production with > 99.99% confidence followed by EDTA (H), K_2_HPO_4_ (C), inoculum size (O), CaCl_2_ (E), and NaCl (K) with > 99.99%, > 99.99%, > 99.99, > 99.99, and 99.99 confidence, respectively.

**Table 3:** Statistical analysis of Plackett–Burman design for polysaccharide production from *A. Al-Azhar MNE ON864448.1*

Factor	Coefficient estimate	Stdized effect	% Contribution
Intercept	0.4896	–	–
A-NaHCO_3_	0.0025	0.0049	0.023
B-NaNO_3_	0.0040	0.008	0.062
C-K_2_HPO_4_	−0.0614	−0.1227	14.3
D-MgSO_4_.7H_2_O	0.0020	0.004	0.015
E-CaCl_2_	0.0544	0.1089	11.29
F-Citric acid	−0.0002	−0.00041	0.00016
G-Ferric ammonium citrate	0.0027	0.0054	0.027
H-EDTA	0.0705	0.14	18.9
J-Trace metal	0.0023	0.0046	0.021
K-NaCl	0.0528	0.105	10.64
L-Temperature	−0.0018	−0.0035	0.0119
M-pH	−0.0013	−0.0025	0.0063
N-working volume	0.0896	0.179	30.64
O-Inoculum size	0.0594	0.118	13.45

To validate the obtained results of the experimental design, analysis of variance (ANOVA) for polysaccharide production was performed and the sum of the square, confidence level, *P*-value, *F*-value, and the mean square is displayed in Additional file 1: Table S2. The probability known as the *P*-value can be used to assess the significance of a model and each variable. A real or significant effect is indicated by a low *P*-value. Model terms are significant when *P* < 0.05 is present. The student’s t-test was used to examine the significance of each variable. Our model is significant, as shown by the *P*-value of < 0.0001 and the model *F*-value of 81.2. The ANOVA analysis demonstrated that working volume (N), with an *F*-value of 349.67, *P-*value of < 0.0001, and confidence level of > 99.99% was found to be a more significant variable, followed by EDTA (H) (*F*-value of 216.73, *P*-value of < 0.0001 and confidence level of > 99.99%), K_2_HPO_4_ (C) (*F*-value of 164.34, *P*-value of < 0.0001 and confidence level of > 99.99%), inoculum size (O) (*F*-value of 153.65, *P*-value of < 0.0001 and confidence level of > 99.98%), CaCl_2_ (E) (*F*-value of 128.91, *P*-value of < 0.0001 and confidence level of > 99.98%), and NaCl (K) (*F*-value of 121.57, *P*-value of 0.0001 and confidence level of 99.99%) are the most effective parameters affecting polysaccharide production. Moreover, it was obvious that among these parameters, only K_2_HPO_4_ (C) exerted a negative effect, whereas the other factors (working volume (N), EDTA (H), inoculum size (O), CaCl_2_ (E), and NaCl (K)) exerted positive effects on cyanobacterial polysaccharide production, which means that the decrease in K_2_HPO_4_ (C) and an increase in working volume (N), EDTA (H), inoculum size (O), CaCl_2_ (E), and NaCl (K) could exert a positive impact on polysaccharide production. While other screened parameters NaHCO_3_ (A), NaNO_3_ (B), MgSO_4_ (D), citric acid (F), trace metal (J), ferric ammonium citrate (G), temperature (L), and pH (M), have no significant influence on the cyanobacterial polysaccharide production from *A. Al-Azhar MNE ON864448.1*.

A higher correlation coefficient (*R*^2^ = 0.9956) value indicated a good correlation between the experimental and predicted values. The coefficient of determination (*R*^2^) value provides a measurement of how much variation in the observed response values can be explained by the experimental variables. The *R*^2^ value is always between 0 and 1. The more accurate the model and stronger the expected response, the closer the *R*^2^ to 1 [70]. The coefficient of determination value (*R*^2^ = 0.9956) showed that 99.56% of the variations in cyanobacterial polysaccharide production can be demonstrated by the independent variables and only 0.44% of the variations are not demonstrated by these variables. As well, a very high adjusted coefficient of determination value (Adj. *R*^2^ = 0.9834) demonstrates the high significance of the model [71]. The Predicted *R*^2^ value of 0.9299 is close to the adjusted *R*^2^ value of 0.9834. This reveals a good correlation between the experimental and predicted values of cyanobacterial polysaccharide synthesis. Adequate Precision measures the signal-to-noise ratio and the ratio greater than 4 is desirable. The ratio of 29.7989 demonstrates an adequate signal and the possibility of this model being navigated to the design space. The coefficient of variation (C.V.%) is a statistical measure of data residual variation relative to the size of the mean or demonstrated dispersion of data points in a data series around the mean. Generally, the lower the value of C.V. the higher the reliability of the experiment. In this study, the lower value of C.V.% (4.37%) indicates a greater accuracy of the performed experiments. The predicted residual sum of squares (PRESS) is a measure of how well the model fits each point in the design. The smaller the PRESS statistics, the better the model fits the data points. The PRESS value is 0.03669. In this experiment, the model gives values of the mean and standard deviation of 0.4896 and 0.0214, respectively. The optimum cyanobacterial polysaccharide synthesis as a function of the independent variables was represented using a first-order polynomial equation. The following regression equation in terms of coded variables was produced after ignoring the insignificant variables.

$${\mathrm{Y}} = 0.489648 + 0.0024576*{\mathrm{A}} + 0.0040446*{\mathrm{B}} + - 0.0613915*{\mathrm{C}} + 0.002034*{\mathrm{D}} + 0.0543734*{\mathrm{E}} + - 0.0002063*{\mathrm{F}} + 0.0027002*{\mathrm{G}} + 0.0705023*{\mathrm{H}} + 0.002349*{\mathrm{J}} + 0.0528022*{\mathrm{K}} + - 0.0017658*{\mathrm{L}} + - 0.0012847*{\mathrm{M}} + 0.0895505*{\mathrm{N}} + 0.0593619*{\mathrm{O}}.$$

where Y is the polysaccharide yield and “A, B, C, D, E, F, G, H, J, K, L, M, N and O” are NaHCO_3_, NaNO_3_, K_2_HPO_4_, MgSO_4_, CaCl_2_, citric acid, ferric ammonium citrate, EDTA, trace metal, NaCl, temperature, pH, working volume and inoculum size, respectively.

#### Adequacy of the model

**Fig. 7:**
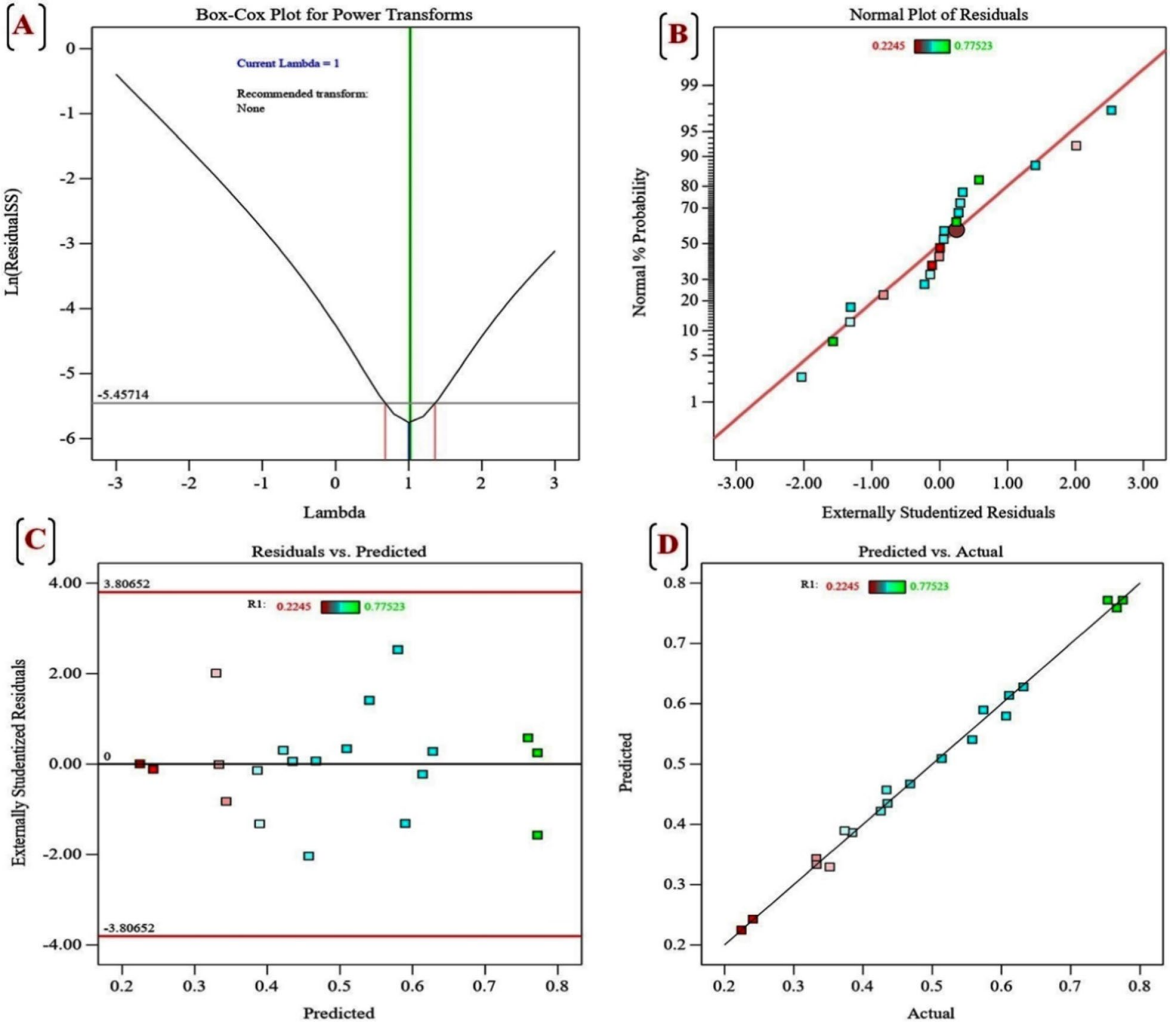
Plackett–Burman diagnostics for *A. Al-Azhar MNE ON864448.1* EPS production, **A** Box–Cox plot of model transformation, **B** Normal probability plot of residuals, **C** plot of internally studentized residuals versus predicted values, and **D** plot of predicted versus actual values for *A. Al-Azhar MNE ON864448.1* EPS production

The normal probability plot (NPP) is represented in Fig. 7B. The normal probability plot of the residuals is a crucial diagnostic tool for identifying and displaying systematic deviations from normality [72]. The NPP of internally studentized residuals displays the points near the diagonal line, indicating that the residuals are normally distributed and that this model fits the experimental data well. The Box–Cox plot is an illustration of a potential best practice for detecting the more effective power transformation to enhance the model. As seen from Fig. 7A, the best lambda value is indicated by the green line (Lambda = 1) and the blue line represents the current transformation (Lambda = 1), while the red lines represent a minimum (0.75) and maximum (1.25) 95% confidence interval values. As the current value of the confidence interval (*λ* = 1) is the same value of the model design (best = 1), this model requires no transformation. The Box–Cox graph showed that the model is in the optimal zone as the blue line falls within the red lines and demonstrates well fitness of the model to the obtained experimental data.

### Face-centered central composite design (FCCD) for optimization of EPS production

The assessment of the Plackett–Burman design displayed that, working volume (X1), EDTA (X2), inoculum size (X3), CaCl_2_ (X4), and NaCl (X5) influenced the cyanobacterial polysaccharide production effectively. So, FCCD was employed to determine the optimum levels of these variables which give maximum production of cyanobacterial polysaccharide and their interactions with each other. Variables with positive impacts on cyanobacterial production have been used at a high level, while the variables that have a negative effect are kept at a low level for further optimization by FCCD. The effect of working volume (X1), EDTA (X2), inoculum size (X3), CaCl_2_ (X4), and NaCl (X5) and their interactions on cyanobacterial polysaccharide production was studied by FCCD at five levels (−2, −1, 0, 1, 2) using 30 runs and the results are presented in Additional file 1: Table S3. According to the experimental results, the cyanobacterial polysaccharide yield ranged from 2.81 to 9.27 mg/mL. The maximum polysaccharide yield was observed in run number 30 with a value of 9.27 mg/mL, where working volume 300 mL of 1000 mL volumetric flask, EDTA 0.002 g/L, inoculum size 7%, CaCl_2_ 0.046 g/L, and NaCl 20 g/L were used. Whereas the minimum polysaccharide yield was obtained in run number 17 with a value of 2.81 mg/mL, working volume 600 mL of 1000 mL volumetric flask, EDTA 0.003 g/L, inoculum size 6%, CaCl_2_ 0.036 g/L, and NaCl 25 g/L were used. In Additional file 1: Table S3 each experimental value of polysaccharide yield was in good correlation with the model’s predictable value.

### Multiple regression analysis and ANOVA

Using the Design Expert 13 software, the experimentally obtained data were conducted for regression analysis and analysis of variance (ANOVA), and the generated results are displayed in Additional file 1: Tables S4–S6. The coefficient of determination (*R*^2^) evaluates the goodness of fit of the designed model. In the present study, the resulting coefficient of determination (*R*^2^) value (0.9839) (Additional file 1: Table S4) indicated that 98.39% of the variations in the polysaccharide production can be explained by the independent variables and only 1.61% of the variations cannot be explained by these variables. The regression model is considered highly correlated when the *R*^2^ value is higher than 0.9. The adjusted determination coefficient (Adj *R*^2^) was found to be 0.9547 which confirm the model significance. The values of the predicted coefficient (Pred *R*^2^) and the Adj *R*^2^ were found to be 0.906 and 0.9547, respectively, which illustrates a very good fit between the theoretically predicted values and the experimentally obtained data by the model [73] and suggested that the model is reliable for polysaccharide production from *A*. *Al-Azhar MNE ON864448.1*. Moreover, the Adeq Precision value was very high (28.0502) revealing that the model can be used to navigate the design space. The lower value of the coefficient of variation % (6.17) suggested that the experimental performance was more accurate and reliable [74]. The obtained PRESS value is 5.76, and the model gives values of the mean and standard deviation of 4.61 and 0.2845; respectively (Additional file 1: Table S4). Additional file 1: Table S4 indicates the interactions between two factors could appear as a synergistic effect (positive coefficient) or an antagonistic effect (negative coefficient). The positive coefficients for X3, X4, X5, X1X3, X2X4, X3X4, and X3X5 indicate that the linear effect of X3, X4, X5, and interaction effects for X1X3, X2X4, X3X4, and X3X5 increase polysaccharide yield, whereas other negative coefficients indicate a decrease in the polysaccharide yield.

Additional file 1: Table S5 illustrates the analysis of variance (ANOVA) values for the quadratic regression model obtained from FCCD employed in the optimization of cyanobacterial polysaccharide production. The *P*-value was calculated to validate the significance of each coefficient and is also necessary for understanding the mutual interactions between the factors. The *P*-values are listed in Additional file 1: Table S5. Values of Prob > *F* (*P*-values) less than 0.05 indicate the significance of model terms while values greater than 0.05 indicate the model terms are not significant. The analysis of variance confirms that the highly significant model is predicted from Fisher’s *F*-test (33.68) and a very low *P*-value < 0.0001. It can be seen that the linear coefficients of working volume (X1), EDTA (X2), inoculum size (X3), CaCl2 (X4), and NaCl (X5), the interactions of X1X4, and X1X5 and quadratic effects of X1, X2, and X5 are significant while the interactions of X1X2, X1X3, X2X3, X2X4, X2X5, X3X4, X3X5 and X4X5 and quadratic effects of (X3) and (X4) is not significant (*P*-value < 0.05) (Additional file 1: Table S5).

The probability values of the coefficient indicated that among the studied five variables working volume (X1), inoculum size (X3), CaCl2 (X4), NaCl (X5), and quadratic effect of working volume (X1) show maximum *P*-value > 0.0001 demonstrating 99.99% of the model affected by these variables. As illustrated in Additional file 1: Table S6, the statistics of the quadratic model summary showed the highest *R*^2^, adj *R*^2^, and pred *R*^2^ of 0.9839, 0.9547, and 0.906; respectively, and a lower standard deviation of 0.2845. The fit summary was confirmed the adequacy and the high significance of the quadratic model with a very low *P*-value < 0.0001. To determine the relationship between the cyanobacterial polysaccharide yield and the independent variables and to determine the optimal concentration of each component involved in cyanobacterial polysaccharide production, the equation of second-order polynomial was obtained to define the predicted cyanobacterial polysaccharide yield (Y) in terms of the independent variables:

$${\mathrm{Y}} = 4.5154 - 1.00582*{\mathrm{X}}1 - 0.26544*{\mathrm{X}}2 + 0.358287*{\mathrm{X}}3 + 0.34834*{\mathrm{X}}4 + 0.388783*{\mathrm{X}}5 - 0.0523385*{\mathrm{X}}1{\mathrm{X}}2 + 0.073062*{\mathrm{X}}1{\mathrm{X}}3 - 0.195954*{\mathrm{X}}1{\mathrm{X}}4 - 0.191207*{\mathrm{X}}1{\mathrm{X}}5 - 0.00249073*{\mathrm{X}}2{\mathrm{X}}3 + 0.000817728*{\mathrm{X}}2{\mathrm{X}}4 - 0.0516018*{\mathrm{X}}2{\mathrm{X}}5 + 0.0916582*{\mathrm{X}}3{\mathrm{X}}4 + 0.0985327*{\mathrm{X}}3{\mathrm{X}}5 + - 0.00186573*{\mathrm{X}}4{\mathrm{X}}5 + 0.660819*{\mathrm{X}}1^{2} - 0.179628*{\mathrm{X}}2^{2} - 0.0696186*{\mathrm{X}}3^{2} - 0.0213876*{\mathrm{X}}4^{2} - 0.258153*{\mathrm{X}}5^{2} ,$$

where Y is the polysaccharide yield and “X1, X2, X3, X4, and X5” are working volume, EDTA, inoculum size, CaCl_2_, and NaCl, respectively.

#### Three-dimensional plots

**Fig. 8:**
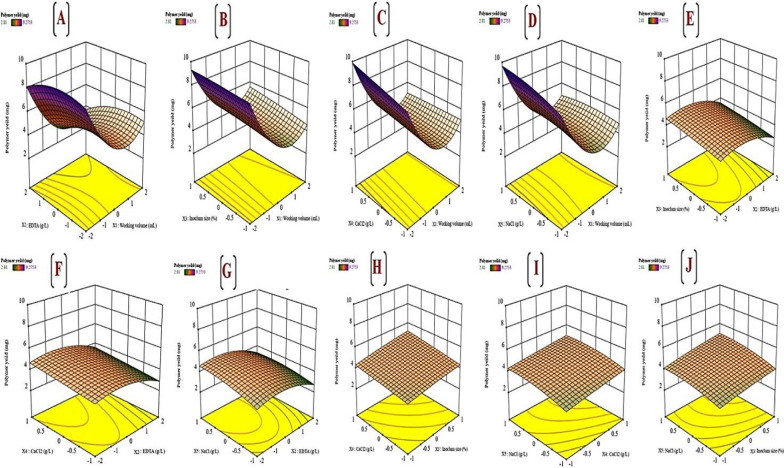
**A–J** 3D plots generated using Design Expert version 13 for Windows software displaying the interaction effects between working volume (X1) and EDTA concentration (X2) by keeping inoculum size (X3), CaCl_2_ (X4), and NaCl (X5) on *A. Al-Azhar MNE ON864448.1* EPS production

The three-dimensional surface graphs and their corresponding contour plots explained the interaction of different studied variables and the optimal levels of each variable involved in cyanobacterial polysaccharide production. Response plotting curves represented the effect of three fixed variables at their optimum levels when the other two variables are varying (Fig. 8A–J). Figure 8A illustrates cyanobacterial polysaccharide production as affected by the working volume (X1) and EDTA concentration (X2) by keeping inoculum size (X3), CaCl_2_ (X4), and NaCl (X5) at the best value. It showed that when the working volume (X1) decreases while EDTA concentration decreases, cyanobacterial polysaccharide production gradually increases. This observation depends on the fact that, EDTA chelates ions necessary for cyanobacterial growth leading to nutrient deficiency. Therefore, our cyanobacterium isolate; *A. Al-Azhar MNE ON864448.1* tends to produce more EPS as a protective strategy [75]. Figure 8B displayed the effect of working volume (X1) and inoculum size (X3) on cyanobacterial polysaccharide production by keeping EDTA concentration (X2), CaCl_2_ (X4), and NaCl (X5) at their optimal value. It displayed that increasing in polysaccharide production was detected as working volume (X1) decreased and inoculum size (X3) increased. The inoculum size should be based on the steady state of each strain; however, this procedure requires a previous understanding of each strain’s growth curve. Although several previous EPS screening studies reported that microalgae are inoculated according to a specific concentration in terms of cell mass, cell number, chlorophyll, or even only a percentage, they have rarely supplied information on inoculum. Our studies displayed that; strong inoculum for a small cultivation volume highlighted the high polysaccharide yield from *A. Al-Azhar MNE ON864448.1*. Figure 8C illustrates the interaction effect of working volume (X1) and CaCl_2_ (X4) on polysaccharide production by keeping EDTA concentration (X2), inoculum size (X3), and NaCl (X5) at their optimal value. It displayed that; maximum production of cyanobacterial polysaccharide was obtained as CaCl_2_ (X4) concentration increased with the working volume decreased. Micronutrients such as CaCl_2_ have been displayed to produce significant effects on EPS production from cyanobacteria [46]. Figure 8D showed the interaction effect of working volume (X1) and NaCl (X5) on polysaccharide production by keeping EDTA concentration (X2), inoculum size (X3), and CaCl_2_ (X4) at their optimal value. The maximum polysaccharide yield was achieved by decreasing working volume (X1) while increasing NaCl concentration (X5). Our findings demonstrate that cyanobacterial strains with slower growth rates may survive in high-salinity environments through the production of exopolysaccharides around the outer cell membrane. Under salt stress (high levels of NaCl or CaCl_2_), cells’ investment in increased exopolysaccharide synthesis by reducing growth rate may be attributed to spending energy for survival rather than replication. An improved exopolysaccharide layer can protect cyanobacterial cells from dehydration by developing a microenvironment around the cell wall that buffers osmotic disequilibrium across the cell membrane, giving a repository for water and reducing the ion influx under hypersaline stress [51, 52]. The interaction effects of EDTA concentration (X2) with other variables [inoculum size (X3), CaCl_2_ (X4), and NaCl (X5)] on cyanobacterial polysaccharide production are represented by Fig. 8E–G. The interaction effect of EDTA concentration (X2) and inoculum size (X3) on the cyanobacterial polysaccharide production shown in Fig. 8E that indicates the moderate polysaccharide yield as the EDTA concentration (X2) decreased with a high or low level of inoculum size (X3) while polysaccharide production was retarded with increasing EDTA concentration (X2) by keeping the working volume (X1), CaCl_2_ (X4), and NaCl (X5) at their optimal value.

Figure 8F demonstrates the moderate polysaccharide yield as the EDTA concentration (X2) decreased with high or low-level concentration of CaCl_2_ (X4) while polysaccharide production was retarded with increasing EDTA concentration (X2) by keeping the working volume (X1), inoculum size (X3), and NaCl (X5) at their optimal value. The same effect was observed with the interaction of EDTA (X2) and NaCl (X5) while keeping the working volume (X1), inoculum size (X3), and CaCl_2_ (X4) at their optimal value as Fig. 8G representing that the moderate polysaccharide yield was achieved as the EDTA concentration (X2) decreased with increasing concentration of NaCl (X5) up to 20 g/L. These observations may be attributed to EDTA chelation activity for Na^+^ ions responsible for increasing cyanobacterial EPS production (salt stress). In this experiment, the interaction effects of inoculum size (X3) and CaCl_2_ (X4) on polysaccharide production at fixed optimal values of working volume (X1), EDTA (X2), and NaCl (X5) as shown in Fig. 8H which represents that moderate polysaccharide yield as increasing the level of both inoculum size (X3) and CaCl_2_ (X4) while at a low level of both variables, the polysaccharide yield was reduced. The same effect of interaction was noticed between inoculum size (X3) and NaCl (X5) on polysaccharide production Fig. 8J. Figure 8I represents the yield of cyanobacterial polysaccharide as affected by the interaction of CaCl_4_ (X4) and NaCl (X5) while keeping the working volume (X1), EDTA (X2), and inoculum size (X3) at optimum value. A low polysaccharide yield was detected at a low level of both variables while a moderate yield was detected at the highest level of CaCl_4_ (X4) and NaCl (X5) while keeping the other variables at their optimal level (X = 0).

### Cytotoxicity and safety of the purified cyanobacterial EPS

The cytotoxic effects were evaluated in the Vero cell line after 72 h of treatment with different* A. Al-Azhar MNE ON864448.1* polysaccharide concentrations. The cytotoxic potential increased in a dose-dependent manner with a cell death rate greater than 50% at concentrations of 200 µg/mL after 72 h of treatment. In detail, the recorded IC_50_ was 151.4 µg/mL for polysaccharide extracted from *A. Al-Azhar MNE ON864448.1*. The antiviral activity was evaluated at a concentration that did not affect cell viability; therefore, it was lower than 100 µg/mL. Microalgae polysaccharides exhibit a wide range of advantageous characteristics when compared to alternative sources. These include their safety, biocompatibility, biodegradability, stability, and versatility. Furthermore, they displayed various biological characteristics such as antioxidant, anti-inflammatory, antitumor, and antimicrobial activities. These activities are closely linked to the molecular weight, sulfate, uronic acids, or charged group content of the polysaccharides [76]. Our results matched the previous studies reported about cyanobacterial polysaccharide toxicity for example A1 and A2 are extracellular sulfated polysaccharides isolated from marine microalga, *and Cochlodinium polykrikoides* didn’t show any adverse effect or cytotoxicity at a concentration of 100 µg/mL [77].

### Antiviral activity of the purified cyanobacterial EPS

The antiviral activities of polysaccharide extracted from* A. Al-Azhar MNE ON864448.1* were examined against enveloped DNA virus (HSV-1, HSV-2), non-enveloped DNA virus (ADV) and RNA virus (Coxsackievirus) by plaque reduction assay in Vero cells line and the obtained results were confirmed by MTT assay. The viruses included in this study were chosen based on their varying degrees of virulence, ranging from severe to mild. Furthermore, these viruses belong to different viral classes to ensure the effectiveness of the purified EPS as an antiviral agent.

#### Virucidal activity of cyanobacterial EPS against Herpesviridae

**Fig. 9:**
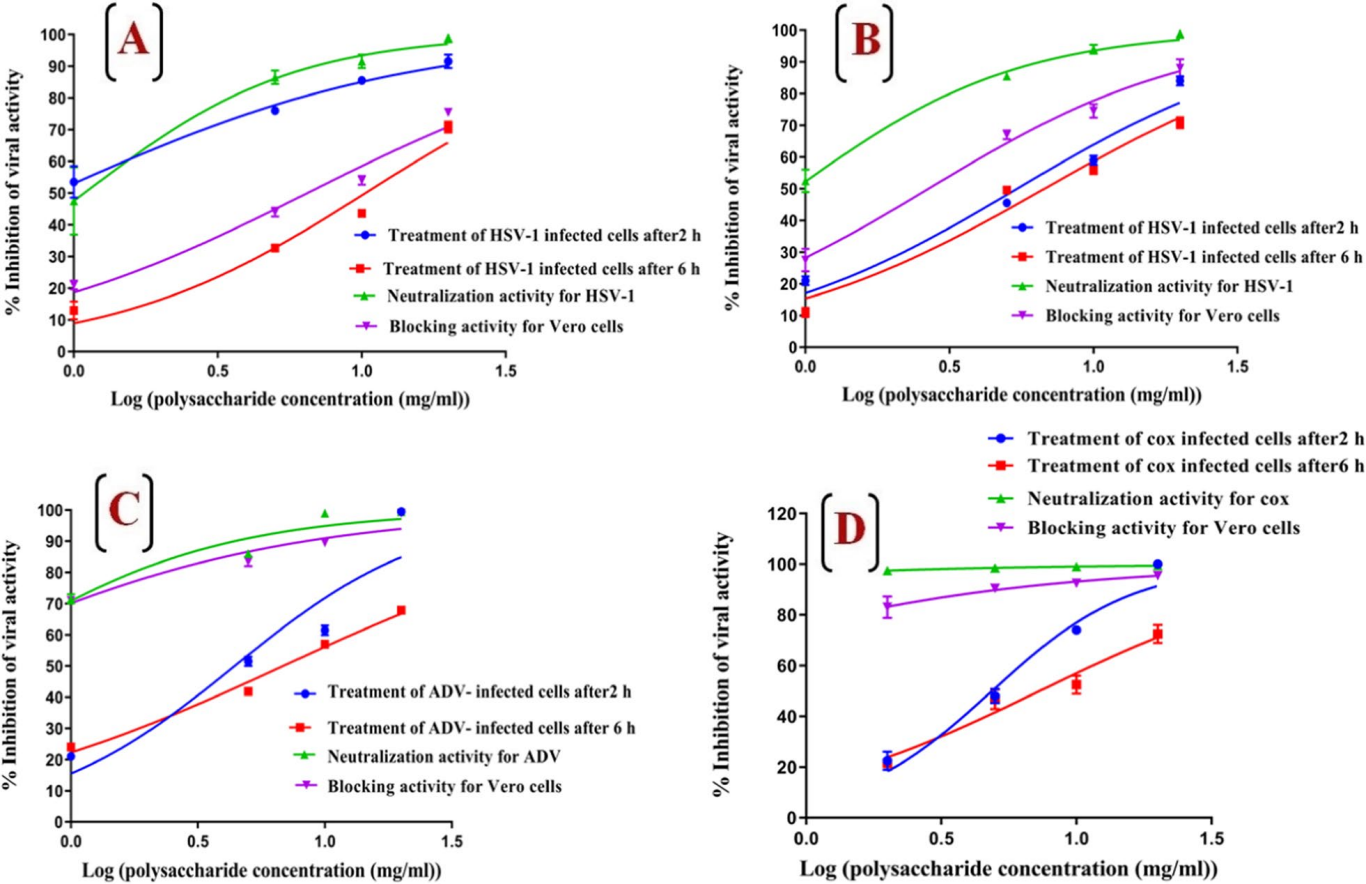
Displaying antiviral activity (neutralization, blocking, and treatment activities) of *A. Al-Azhar MNE ON864448.1* EPS. **A** Non-linear regression analysis of cyanobacterial EPS antiviral activity against HSV-1 using graph pad prism software version 9. **B** Non-linear regression analysis of cyanobacterial EPS antiviral activity against HSV-2 using graph pad prism software version 9. **C** Non-linear regression analysis of cyanobacterial EPS antiviral activity against ADV using graph pad prism software version 9. **D** Non-linear regression analysis of cyanobacterial EPS antiviral activity against Coxsackievirus using graph pad prism software version 9

Viruses are obligate intracellular pathogens, and their infections are complicated involving several steps. To understand the polysaccharide viral target, it was added at different times during viral infection. Initially, during the extracellular infection phase, a neutralization assay or virus–pretreatment assay was applied to examine the antiviral activity of cyanobacterial polysaccharide after 2 h-incubation with TCID of HSV-1 at different polysaccharide concentrations. Cytopathic reduction assay revealed that polysaccharide extracted from *A. Al-Azhar MNE ON864448.1* significantly inactivated HSV-1 and HSV-2 virus particles in a dose-dependent manner and recorded HSV-1 inhibition rate of 88 ± 1.4, 93.5 ± 2.1 and 99 ± 1.3% and HSV-2 inhibition rate of 85.5 ± 0.3, 94.5 ± 0.55, and 99 ± 0.1% at a final cyanobacterial polysaccharide concentration of 5, 10 and 20 µg/mL, respectively (Table 4 and Fig. 9). These results indicated that cyanobacterial polysaccharides could be effective in the extracellular infection phase, altering the viral structure. To confirm these results 100 µl of HSV-1/HSV-2-polysaccharide mixture was used for Vero cell infection for 2 h and cell survival was determined after 72 h of incubation using MTT colorimetric assay. The obtained MTT results highly matched with cytopathic reduction assay revealing that polysaccharide extracted from *A. Al-Azhar MNE ON864448.1* displayed neutralization activities of 86.5 ± 2.1, 91.5 ± 1.1 and 99 ± 0.55% against HSV-1 and 85 ± 0.7, 94 ± 1.1, and 99 ± 0.4% against HSV-2 at polysaccharide concentrations of 5, 10, and 20µg/mL, respectively (Table 4 and Fig. 9). Five viral glycoproteins, namely gB, gC, gD, gH, and gL, have been implicated in the process of viral entry [78, 79]. All these glycoproteins, except for gC, play an essential role in viral entry. The initial interaction, or binding to cells, is facilitated through the interactions between gC and/or gB with heparan sulfate proteoglycans (HSPGs). Similar to the attachment process, membrane fusion requires involvement from cellular receptors. Various receptors for gD, such as nectin-1 and -2, herpesvirus entry mediator (HVEM), and 3-O sulfated heparan sulfate (3-O HS), have been discovered [78, 80]. Furthermore, cyanobacterial EPS can hinder the activity of type 1 and type 2 herpes simplex virus (HSV) through ionic interaction. This interaction occurs between the positively charged external glycoproteins present on the enveloped virus surface and the negatively charged cyanobacterial sulfated EPS. Carrageenan and its oligosaccharide derivatives bind to the glycoprotein on the virus surface, leading to denaturation and inactivation of the HSV glycoprotein. As a result, viral adsorption and replication within the host cell are inhibited [81].

**Table 4:** (%) Antiviral activity of cyanobacterial EPS against different viruses using plaque assay

Virus	Concentration (µg/mL)	Treatment	Neutralization	Blocking
HSV-1	5	76.9 ± 0.14	88 ± 1.4	44.7 ± 0.88
	10	86.5 ± 0.7	93.5 ± 2.1	54.5 ± 1.1
	20	91.5 ± 1.83	99 ± 1.3	77.2 ± 1.3
	EC_50_ (µg/mL)	0.8424	1.088	6.477
	SI (IC_50_/EC_50_)	179.72	140.185	23.65
HSV-2	5	54.79 ± 1.7	85.5 ± 0.3	76.3 ± 0.7
	10	59.02 ± 1.3	94.5 ± 0.55	78.5 ± 1
	20	85 ± 0.72	99 ± 0.1	88 ± 0.5
	EC_50_ (µg/mL)	5.433	0.9233	2.676
	SI	27.866	163.977	56.57
ADV	5	51.5 ± 0.14	72.12 ± 1.3	71 ± 0.55
	10	61.94 ± 0.07	85.22 ± 0.52	83.5 ± 2.1
	20	99.6 ± 0.89	99.7 ± 0.13	89.7 ± 0.8
	EC_50_ (µg/mL)	4.415	0.3664	0.2605
	SI	34.29	413.66	581.19
Coxsackie virus	5	47 ± 1.4	98.5 ± 1	91.5 ± 1.2
	10	73.5 ± 0.7	99.2 ± 1.5	94 ± 0.5
	20	99 ± 2.1	99.7 ± 0.9	95.2 ± 1.1
	EC_50_ (µg/mL)	4.872	0.005669	0.1558
	SI	31.075	30,280	971.75

#### Blocking activity of cyanobacterial EPS against Herpesviridae

On the other hand, the ability of cyanobacterial EPS to block viral receptor entry was detected after pre-incubating Vero cells with different cyanobacterial polysaccharide concentrations, which were subsequently infected with TCID_50_ of HSV-1 and HSV-2. Cytopathic reduction results indicated that polysaccharide extracted from *A. Al-Azhar MNE ON864448.1* compete HSV-1 and HSV-2 on cellular receptors and displayed protecting activities of 44.7 ± 0.88, 54.5 ± 1.1 and 77.2 ± 1.3% for Vero cells against HSV-1 and 76.3 ± 0.7, 78.5 ± 1 and 88 ± 0.5 against HSV-2 at final concentrations of 5, 10, and 20 µg/mL, respectively. MTT results confirmed these results and indicated protection activities of 43 ± 0.9, 54.2 ± 0.17 and 75.5 ± 0.61% against HSV-1 and 75 ± 0.4, 74.5 ± 0.6 and 88 ± 0.32 against HSV-2 at final concentrations of 5, 10, and 20 µg/mL, respectively (Table 4 and Fig. 9). From these results we assume that cyanobacterial polysaccharides extracted from *A. Al-Azhar MNE ON864448.1* have an affinity for host cell receptor; heparan sulfate moiety (HS) of heparan sulfate proteoglycan (HSPGs) and competes for herpesviruses glycoproteins on cellular receptors. Certain sulfated marine polysaccharides can engage with receptors on viruses, thereby obstructing their interaction with the surface of the host cell. Alternatively, these polysaccharides can have a direct interaction with the viral particles themselves, thereby hindering virus infection. Multiple studies have provided evidence that the negatively charged sulfate groups found in carrageenan can interact with viruses by concealing the positive charge of their receptors [82].

#### Effect of cyanobacterial EPS on Herpesviridae propagation or replication

The cytopathic reduction results displayed that polysaccharide extracted from *A. Al-Azhar MNE ON864448.1* reduced viral plaque count in HSV-1 and HSV-2 infected cells in a dose-dependent manner at an early stage of infection. The extracted cyanobacterial EPS significantly inhibited HSV-1 and HSV-2 replication after 2 h infection with relative inhibitory activities of 76.9 ± 0.14, 86.5 ± 0.7, and 91.5 ± 1.83% for HSV-1 and 54.79 ± 1.7, 59.02 ± 1.3 and 85 ± 0.72% for HSV-2 at cyanobacterial polysaccharide final concentrations of 5, 10, and 20 µg/mL, respectively. Also, the obtained results from the colorimetric MTT assay confirmed the outcome of plaque assay and cyanobacterial polysaccharide inhibited viral replication after 2 and 6 h of HSV1 and HSV-2 infection. While MTT results displayed that, after 6 h of viral infection, cyanobacterial polysaccharide inhibited HSV-1 and HSV-2 replication with relative inhibitory activities of 32.7 ± 0.42, 43.6 ± 0.56 and 70.8 ± 1.69%, for HSV-1 and 49.56 ± 0.62, 56.04 ± 1.3 and 72.1 ± 1.2% for HSV-2 at cyanobacterial polysaccharide final concentrations of 5, 10, and 20 µg, respectively (Table 4 and Fig. 9). While cyanobacterial polysaccharides at different concentrations failed to inhibit HSV-1 and HSV-2 replication when added after 24 h of viral infection confirmed that polysaccharides extracted from *A. Al-Azhar MNE ON864448.1* exert their antiviral activity at an early stage of viral replication during intracellular infection phase. This cyanobacterial EPS may bind to an allosteric site of the viral capsid, thereby inhibiting the process of the virus uncoating within the host cell. Additionally, the EPS indirectly obstructs viral infection and expedites the elimination of the virus by triggering the activation of NK cells and stimulating factors of the antiviral immune response [83]. Several publications displayed that cyanobacterial polysaccharides are potent antiviral agents having different antiviral mechanisms. The acidic polysaccharide nostoflan isolated from the blue-green algae *N. flagelliforme* has shown antiviral efficacy against viruses that employ carbohydrates as cellular receptors. It has strong antiviral action against HSV-1, HSV-2, human cytomegalovirus, and influenza A virus. It suppresses the earliest stage of viral infection, including virus binding and internalization [84]. As well, calcium spirulan (Ca-SP) is obtained from hot water extracts of *Spirulina platensis* and possesses antiviral activity against HSP-1, HSV-1, and HIV-1. Even at low doses, it inhibits viral entrance into host cells and syncytium formation [12], *Navicula directa* is an important source of sulfated polysaccharides called naviculan which consists of mannose, xylose, fucose, galactose, and rhamnose sugars [85]. It exhibited unique antiviral activity against HSV-1, HSV-2, and influenza A viruses. It prevents fusion between HIV gp160–expressing HeLa cell line and cells expressing the CD4 receptor [86].

### Antiviral activity of EPS against Adenovirus (ADV)

#### Virucidal activity of cyanobacterial EPS against ADV

The results of the cytopathic reduction and MTT assays revealed that polysaccharides extracted from *A. Al-Azhar MNE ON864448.1* significantly neutralized ADV particles and consequently inactivated their infectivity. The neutralization activity of cyanobacterial polysaccharide for ADV was determined using the cytopathic reduction assay and results demonstrated that polysaccharide extracted from *A. Al-Azhar MNE ON864448.1* is a potent virucidal agent for ADV as it reduced the plaques count in a dose-dependent manner with viral neutralization activity equaled 72.12 ± 1.3, 85.22 ± 0.52 and 99.7 ± 0.13% at polysaccharide final concentrations of 5, 10, and 20 µg, respectively. The outcome of cytopathic reduction assay was confirmed by MTT assay and recorded viral inhibition rate of 71.32 ± 2.4, 86.42 ± 0.82, and 99 ± 0.029% at final cyanobacterial polysaccharide concentrations of 5, 10, and 20 µg/mL, respectively (Table 4 and Fig. 9). The antiviral potential of EPS obtained from *A. Al-Azhar MNE ON864448.1* is believed to be attributed to its capacity to bind to ADV particles and disrupt their structure. Bacterial exopolysaccharides derived from *Lactobacillus* sp. exhibit noteworthy antiviral properties owing to their ability to degrade viral particles, diminish virus titers, impede viral DNA replication, and facilitate the release of infectious virus particles [87].

#### Blocking the activity of cyanobacterial EPS against ADV

On the other hand, the results of both cytopathic reduction and MTT assays indicated that the addition of the polysaccharide extracted from *A. Al-Azhar MNE ON864448.1* to Vero cells for 2 h before ADV infection (pre-treatment) protects the cells from ADV infection at any tested concentration and displayed blocking activity of 71 ± 0.55, 83.5 ± 2.1, and 89.7 ± 0.8% (Table 4) Viral internalization step is very specific and selective process. ADV enters the host cell through two mechanisms: cell-mediated receptor entry and receptor-mediated endocytosis. Cell-mediated receptor mechanisms involve two steps. The initial step involves the attachment of viral penton fiber protein with the Coxsackie virus Adenovirus Receptors (CAR) family’s receptors. The secondary step involves the attachment of the viral penton base (Arginine glycine aspartate motif) with the host cell’s α V integrin. The viral endocytosis process occurs through clathrin-coated pits (CCPS) of the host cell membrane as viral penton base–α V integrin complex triggers AVD endocytosis. These results suggested that polysaccharides extracted from *A. Al-Azhar MNE ON864448.1* have an affinity for host cell receptors responsible for AVD internalization and obstruct viral entry.

#### Effect of cyanobacterial EPS on ADV propagation or replication on Vero cells

During the intracellular phase, a polysaccharide extracted from *A. Al-Azhar MNE ON864448.1* was added after 2 h of ADV infection, and the results of cytopathic reduction assay displayed that cyanobacterial polysaccharide significantly reduced viral plaque count in ADV-infected cells after 2 h in a dose-dependent manner with relative inhibitory activities of 51.5 ± 0.14, 61.94 ± 0.07 and 99.6 ± 0.89% at final cyanobacterial polysaccharide concentrations of 5, 10, and 20 µg/mL, respectively. As well, the obtained results from the colorimetric MTT assay was confirmed to those obtained from plaque assay when used for treating ADV–infected cells recording inhibition rate of 49.2 ± 0.14, 60.88 ± 0.26 and 97.34 ± 1.1% when added after 2 of infection and 40.4 ± 1.2, 55.1 ± 2.3 and 66.34 ± 1.1% when added after 6 h of infection at final concentrations of 5, 10, and 20 µg/mL, respectively (Table 4 and Fig. 9). ADVs can undergo replication in both dividing and non-dividing cells, with the replication process typically lasting around 24–36 h [88]. The infection cycle of ADV can be divided into two distinct phases. The initial or “early” phase involves the entry of the virus into the host cell, including attachment and internalization, as well as the entry of the viral genome into the nucleus and the expression of early genes. Subsequently, in the second or “late” phase, the transcription and translation of late genes occur, utilizing newly generated genomes as a template. Capsid assembly, virion maturation, and release then follow suit. It is estimated that approximately 105 virus particles are released from each infected cell [89]. The findings of the study demonstrate that the cyanobacterial EPS obtained from *A. Al-Azhar MNE ON864448.1* effectively hindered the ADV infection when introduced at a post-infection time of 6 h. This suggests that the EPS may potentially impede the early phase of the replication process, either by interfering with the viral genome or by inhibiting the expression of early genes. This documentary is the first documentary report about cyanobacterial activity against adenovirus; the previously reported cyanobacterial polysaccharide displayed a wide range of antiviral activity with low IC_50_. Sulfated polysaccharides (ASWPH) extracted from *Aphanothece sacrum* exhibited antiviral activity against HSV-2 and Inf A (H1N1) by inhibiting viral adsorption with IC50 equaled 0.32–1.2 µg/mL[90]. Calcium Spirulan (Ca-SP) extracted from *S. platensis* inhibited replication and penetration of HSV-1, HCMV, MeV, MuV, InfA, HIV-1 with EC50 = 0.92–23 µg/mL [91, 92].

### Antiviral activity of cyanobacterial EPS against Coxsackievirus

#### Virucidal activity of cyanobacterial EPS against Coxsackievirus (A16)

The same addition mechanism was followed while examining the antiviral activity of extracted cyanobacterial polysaccharide against positive sense, single-strand RNA virus; *and Coxsackievirus*. The polysaccharide extracted from *A. Al-Azhar MNE ON864448.1* significantly neutralized Coxsackievirus particles and subsequently inactivated its infectivity. The cytopathic reduction assay was employed to test the cyanobacterial polysaccharide’s capacity to neutralize Coxsackievirus. The results indicated that the polysaccharide extracted from *A. Al-Azhar MNE ON864448.1* is a potent virucidal agent for Coxsackievirus as it reduced the plaque count in a dose-dependent manner with viral neutralization activity equal to 98.5 ± 1, 99.2 ± 1.5, and 99.7 ± 0.9% at polysaccharide final concentrations of 5, 10, and 20 µg/mL, respectively. The results of the cytopathic reduction experiment were validated by the MTT assay, which showed viral inhibition rates of 98 ± 0.2, 98.5 ± 2.1, and 99.5 ± 0.3% at final concentrations of cyanobacterial polysaccharide of 5, 10, and 20 µg/mL (Table 4 and Fig. 9). The in vitro antiviral test showed that EPS extracted from *A. Al-Azhar MNE ON864448.1* is a potent virucidal agent for Coxsackievirus. As previously documented, the presence of the sulfuric acid group plays a vital role in the virucidal activity of polysaccharides [93]. This group can supply or enhance the virucidal properties of a polysaccharide through its interaction with viral proteins. This interaction may be attributed to the attractive forces between the negatively charged sulfate group and the positively charged amino groups found on the surface of viral proteins [94].

#### Blocking activity of cyanobacterial EPS against Coxsackievirus (A16)

The addition of the polysaccharide extracted from *A. Al-Azhar MNE ON864448.1* to Vero cells for 2 h before Coxsackievirus infection (pre-treatment) could inhibit Coxsackievirus infection in a dose-dependent manner with inhibition rates 91.5 ± 1.2, 94 ± 0.5, and 95.2 ± 1.1% at final concentrations of 5, 10, and 20 µg/mL, respectively. While the MTT assay recorded inhibition rates of 90.5 ± 0.7, 92 ± 1, and 95.5 ± 0.4% when cyanobacterial polysaccharide was added before 2 h of Coxsackievirus infection at a final concentration of 5, 10, and 20 µg/mL, respectively (Table 4 and Fig. 9). The results suggest that the cyanobacterial EPS shows a preference for host cell receptors that are responsible for the uptake of Coxsackievirus and inhibits the entry of the viral particles.

#### Effect of cyanobacterial EPS on Coxsackievirus (A16) propagation or replication

During the intracellular stage, the outcomes of the cytopathic reduction test presented that cyanobacterial polysaccharide significantly decreased viral plaque count in Coxsackievirus-infected cells in a dose-dependent manner with relative inhibitory activities of 47 ± 1.4, 73.5 ± 0.7, and 99 ± 2.1% when added after 2 h of infection at final cyanobacterial polysaccharide concentrations of 5, 10, and 20 µg/mL, respectively. Additionally, the results from the colorimetric MTT assay were validated with those from the plaque assay and displayed inhibition rates of 48.2 ± 0.14, 74.88 ± 0.26, and 99.34 ± 1.1% when added after 2 h of Coxsackievirus infection and 45.4 ± 1.2, 52.1 ± 2.3 and 71.34 ± 1.1% after 6 h of Coxsackievirus infection at a concentration of 5, 10, and 20 µg/mL, respectively (Table 4 and Fig. 9). The findings indicated that cyanobacterial EPS might hinder the replication of viruses by either interacting with the uncoating stage or impeding the viral replication enzymes. As previously documented, numerous marine polysaccharides can impede the process of virus transcription and replication after they enter the host cells, either by obstructing the replication enzymes or by preventing the synthesis of proteins from messenger RNA within the host cell [95]. Cyanobacterial EPS (calcium spirulan (Ca-SP)), exhibited its antiviral activities in a wide range of viruses, including enveloped and non-enveloped viruses such as HSV-1, HIV-1, influenza A, Human CytoMegalo Virus (HCMV), measles, coxsackie virus, mumps, and polioviruses [12, 96]. This polysaccharide possesses the capability to develop as an alternative antiviral medication to hinder the emergence of drug-resistant strains as calcium spirulan (Ca-SP) that is extracted from *S. platensis* and has been formulated into a microalgal cream, effectively impeding the recurrence of HSV-1 [15]. Finally, Non-linear regression analysis demonstrated that purified EPS from *A. Al-Azhar MNE ON864448.1* is a potent antiviral agent and exerts its antiviral activity when added at different stages of viral replication. Moreover, the low EC_50_ value of 0.005669 and high selective index (SI) of 30,280 indicate that the Coxsackie virus is the most inhibited virus by cyanobacterial EPS especially when incubated for 2 h before infection (Table 4). The limited number of developed drugs is attributed to the diversity of viral species, resulting in a dearth of broad-spectrum antiviral medications akin to those available for bacterial infections. Furthermore, the emergence of escape mutants further complicates the process. Existing antiviral treatments focus on various stages of viral infection, encompassing the attachment and entry into the host cell, dismantling and replication of genetic material, and the assembly and release of new viral particles [97]. However, due to the close interplay between viral mechanisms and the cellular microenvironment, these antiviral agents often impede not only viral infection but also impact the metabolic processes of the host. The development of effective antiviral therapeutics faces several challenges, including the inherent variability of viral genomes (such as the high mutation and recombination rates in RNA viruses), leading to the rapid emergence of resistance against currently employed antiviral agents [98]. Our results displayed that cyanobacterial EPS obtained from *A. Al-Azhar MNE ON864448.1* inhibited a wide range of viruses, including HSV-1, HSV-2, ADV, and Coxsackievirus A16 at different stages of viral infection making a promising antiviral agent which required great efforts to be approved for viral medication or as vaccine adjuvant. As several polysaccharides exhibit diverse immunomodulatory properties, including the activation of natural killer (NK) cells, the maturation of dendritic cells, and the enhancement of cytotoxic lymphocyte function signifying their possible application as vaccine adjuvant [11].

## Conclusion

In conclusion, we successfully purified a novel EPS from a newly isolated halo-tolerant cyanobacterium, *A. Al-Azhar MNE ON864448.1*. Our cyanobacterium isolate produced a higher level of EPS (810 ± 0.01 mg L^−1^ at 22nd of a cyanobacterial growth phase) than any other reported cyanobacteria under non-optimized conditions. The DEAE-52 cellulose ion exchange chromatography yielded 83.75% of purified cyanobacterial EPS indicating the effectiveness of this technique in EPS purification. Different chemical analyses including FTIR, TGA, and GC/MS indicated that this EPS is a sulfated heteropolysaccharide composed of ten different types of monosaccharide units. Statistical optimization using the Plackett–Burman design displayed that, working volume (X1), EDTA (X2), inoculum size (X3), CaCl_2_ (X4), and NaCl (X5) influenced *A. Al-Azhar MNE ON864448.1* EPS production effectively. While the central composite design showed that the maximum polysaccharide yield was 9.27 mg/mL, when working volume 300 mL of 1000 mL volumetric flask, EDTA 0.002 g/L, inoculum size 7%, CaCl_2_ 0.046 g/L, and NaCl 20 g/L were applied. Our study reported that *A. Al-Azhar MNE ON864448.1* EPS interfered with HSV-1, HSV-2, ADV, and Coxsackievirus life cycle and inhibited their infection with EC_50_ ranging from 6.477 to 0.005669 µg/mL. Moreover, the low EC_50_ value of 0.005669 and high selective index (SI) of 30,280 indicate that the Coxsackie virus is the most inhibited virus by cyanobacterial EPS especially when incubated for 2 h before infection. This EPS may be an alternative therapy for different viral infections. In the future, the development of *A. Al-Azhar MNE ON864448.1* EPS as an antiviral medication requires more pharmaceutical investigations and experimental evidence is required to analyze their antiviral activity. We are looking forward to exploring the efficacy of this EPS as a carrier for other antiviral drugs or adjuvant for antiviral vaccines. Although further investigations are required, our findings opened new prospective opportunities to employ natural compounds for the development of innovative treatments in competing against different viruses.

## Supplementary Information

**Additional file 1. Table S1.** The effect of fourteen independent variables with coded levels on EPS production from A. Al-Azhar MNE ON864448.1 using Plackett–Burman experimental design. **Table S2.** Regression statistics and analysis of variance (ANOVA). **Table S3.** FCCD showing cyanobacterial polysaccharide production by the selected strain as influenced by the most significant variables. **Table S4.** FCCD regression statistics, regression coefficients of second order polynomial model for cyanobacterial polysaccharide production. **Table S5.** Analysis of variance (ANOVA) for the quadratic regression model obtained from FCCD for cyanobacterial polysaccharide production. **Table S6.** Displayed the fit summary of FCCD for cyanobacterial EPS production.

## Data Availability

No datasets were generated or analysed during the current study.
