# Supplementary material for: Characterization and optimization of exopolysaccharide extracted from a newly isolated halotolerant cyanobacterium, Acaryochloris Al-Azhar MNE ON864448.1 with antiviral activity
Source: Microb Cell Fact. 2024 Apr 22;23:117. doi: 10.1186/s12934-024-02383-4 (PMC11034128; doi:10.1186/s12934-024-02383-4)
Supplement: Supplementary file 1 — Additional file 1. Table S1. The effect of fourteen independent variables with coded levels on EPS production from A. Al-Azhar MNE ON864448.1 using Plackett–Burman experimental design. Table S2. Regression statistics and analysis of variance (ANOVA). Table S3. FCCD showing cyanobacterial polysaccharide production by the selected strain as influenced by the most significant variables. Table S4. FCCD regression statistics, regression coefficients of second order polynomial model for cyanobacterial polysaccharide production. Table S5. Analysis of variance (ANOVA) for the quadratic regression model obtained from FCCD for cyanobacterial polysaccharide production. Table S6. Displayed the fit summary of FCCD for cyanobacterial EPS production. [file 12934_2024_2383_MOESM1_ESM.pdf]

Characterization and optimization of exopolysaccharide extracted from a newly isolated halotolerant cyanobacterium, *Acaryochloris Al-Azhar MNE ON864448.1* with antiviral activity

Mabroka H. Saad[a,b], Nagwa M. Sidkey[b], Esmail M. El-Fakharany[a,c,*]

[a]Protein Research Department, Genetic Engineering and Biotechnology Research Institute (GEBRI), City of Scientific Research and Technological Applications (SRTA-City), New Borg EL Arab, Alexandria, Egypt.

[b]Botany & Microbiology Department, Faculty of Science, Al Azhar University (Girls Branch), Nasr City, Egypt. [c]Pharmaceutical and Fermentation Industries Development Centre (PFIDC), The City of Scientific Research and Technological Applications (SRTA-City), Borg Al-Arab, Alexandria, Egypt.

[*]Corresponding author: Esmail M. El-Fakharany (email: esmailelfakharany@yahoo.co.uk).

Table S1. The effect of fourteen independent variables with coded levels on EPS production from *A. Al-Azhar MNE ON864448.1* using Plackett–Burman experimental design

Std	Run	A: $NaHCO_3$	B: $NaNO_3$	C: K_2HPO_4	D: $MgSO_4.7H_2O$	E: $CaCl_2$	F: Citric acid	G: Ferric ammonium citrate	H: EDTA	J: Trace metal	K: NaCl	L: Temperature	M: pH	N: working volume	O: Inoculum size	P: Dummy 1	Q: Dummy 2	R: Dummy 3	S: Dummy 4	T: Dummy 5	Polysaccharide yield (mg/ml)		Residuals
		g/L	g/L	g/L	g/L	g/L	g/L	g/L	g/L	mL	g/L	°C		mL	%	-	-	-	-	-	Actual values	Predicted values	
9	1	1	-1	-1	-1	-1	1	1	-1	1	1	-1	-1	1	1	1	1	-1	1	-1	0.6315	0.6279	0.0036
13	2	1	-1	1	-1	1	-1	-1	-1	-1	1	1	-1	1	1	-1	-1	1	1	1	0.6109	0.6138	-0.0029
20	3	-1	-1	-1	-1	-1	-1	-1	-1	-1	-1	-1	-1	-1	-1	-1	-1	-1	-1	-1	0.2245	0.2244	0.0001
10	4	-1	1	-1	-1	-1	-1	1	1	-1	1	1	-1	-1	1	1	1	1	-1	1	0.57368	0.5898	-0.0161
5	5	-1	1	1	-1	1	1	-1	-1	1	1	1	1	-1	1	-1	1	-1	-1	-1	0.4355	0.4347	0.0008
18	6	-1	-1	1	1	1	1	-1	1	-1	1	-1	-1	-1	-1	1	1	-1	1	1	0.434	0.4570	-0.0230
16	7	1	1	1	1	-1	1	-1	1	-1	-1	-1	-1	1	1	-1	1	1	-1	-1	0.55761	0.5405	0.0171
7	8	-1	-1	-1	1	1	-1	1	1	-1	-1	1	1	1	1	-1	1	-1	1	-1	0.77523	0.7720	0.0032
15	9	1	1	1	-1	1	-1	1	-1	-1	-1	-1	1	1	-1	1	1	-1	-1	1	0.37332	0.3895	-0.0162
1	10	1	1	-1	-1	1	1	1	1	-1	1	-1	1	-1	-1	-1	-1	1	1	-1	0.6066	0.5798	0.0268
2	11	-1	1	1	-1	-1	1	1	1	1	-1	1	-1	1	-1	-1	-1	-1	1	1	0.42572	0.4218	0.0039
19	12	1	-1	-1	1	1	1	1	-1	1	-1	1	-1	-1	-1	-1	1	1	-1	1	0.33309	0.3332	-0.0001
8	13	-1	-1	-1	-1	1	1	-1	1	1	-1	-1	1	1	1	1	-1	1	-1	1	0.753247	0.7720	-0.0188
6	14	-1	-1	1	1	-1	1	1	-1	-1	1	1	1	1	-1	1	-1	1	-1	-1	0.38453	0.3864	-0.0018
4	15	1	1	-1	1	1	-1	-1	1	1	1	1	-1	1	-1	1	-1	-1	-1	-1	0.766379	0.7589	0.0075
17	16	-1	1	1	1	1	-1	1	-1	1	-1	-1	-1	-1	1	1	-1	1	1	-1	0.351948	0.3291	0.0228
3	17	1	-1	1	1	-1	-1	1	1	1	1	-1	1	-1	1	-1	-1	-1	-1	1	0.467864	0.4670	0.0009
12	18	-1	1	-1	1	-1	-1	-1	-1	1	1	-1	1	1	-1	-1	1	1	1	1	0.513549	0.5092	0.0044
11	19	1	-1	1	-1	-1	-1	-1	1	1	-1	1	1	-1	-1	1	1	1	1	-1	0.241173	0.2427	-0.0015
14	20	1	1	-1	1	-1	1	-1	-1	-1	-1	1	1	-1	1	1	-1	-1	1	1	0.33262	0.3432	-0.0106
Low actual (−1)		0.7	0.5	0.02	0.04	0.016	0.003	0.003	0.0005	0.5	0	22	7	400	3	-	-	-	-	-	-	-	-
High actual (1)		3.4	3	0.06	0.1	0.056	0.009	0.009	0.001	1.5	10	30	10	600	7	-	-	-	-	-	-	-	-

Table S2. Regression statistics and analysis of variance (ANOVA).

Source	Sum of Squares	Df	Mean Square	F-value	p-value	Confidence level (%)
**Model**	0.5214	14	0.0372	81.20	< 0.0001	>99.99
A-$NaHCO_3$	0.0001	1	0.0001	0.2634	0.6297	37.03
B-$NaNO_3$	0.0003	1	0.0003	0.7133	0.4369	56.31
C-K_2HPO_4	0.0754	1	0.0754	164.34	< 0.0001	>99.99
D-$MgSO_4.7H_2O$	0.0001	1	0.0001	0.1804	0.6887	31.13
E-$CaCl_2$	0.0591	1	0.0591	128.91	< 0.0001	>99.99
F-Citric acid	8.512E-07	1	8.512E-07	0.0019	0.9673	3.27
G-Ferric ammonium citrate	0.0001	1	0.0001	0.3179	0.5972	40.28
H-EDTA	0.0994	1	0.0994	216.73	< 0.0001	>99.99
J-Trace metal	0.0001	1	0.0001	0.2406	0.6445	35.55
K-NaCl	0.0558	1	0.0558	121.57	0.0001	99.99
L-Temperature	0.0001	1	0.0001	0.1360	0.7274	27.26
M-pH	0.0000	1	0.0000	0.0720	0.7992	20.08
N-working volume	0.1604	1	0.1604	349.67	< 0.0001	>99.99
O- Inoculum size	0.0705	1	0.0705	153.65	< 0.0001	>99.99
**Residual**	0.0023	5	0.0005			
**Cor. Total**	0.5237	19				
**Std. Dev.**	0.0214		**R^2**	0.9956		
**Mean**	0.4896		**Adjusted R^2**	0.9834		
**C.V. %**	4.37		**Predicted R^2**	0.9299		
**PRESS**	0.03669		**Adeq Precision**	29.7989		

Table S3. FCCD showing cyanobacterial polysaccharide production by the selected strain as influenced by the most significant variables.

Std	Run	X1: Working volume	X2: EDTA	X3: Inoculum size	X4: $CaCl_2$	X5: NaCl	Polysaccharide yield (mg/ml)		Residuals
		mL	g/L	%	g/L	g/L	Actual values	Predicted values	
23	1	0	0	0	-2	0	3.84706	3.73	0.1139
13	2	-1	-1	1	1	1	7.48235	7.52	-0.0351
31	3	0	0	0	0	0	3.94706	4.52	-0.5683
32	4	0	0	0	0	0	4.79324	4.52	0.2778
19	5	0	-2	0	0	0	4.3529	4.33	0.0251
5	6	-1	-1	1	-1	-1	4.74706	4.79	-0.0399
18	7	2	0	0	0	0	5.26471	5.15	0.1177
9	8	-1	-1	-1	1	-1	5.4706	5.50	-0.0287
25	9	0	0	0	0	-2	2.82353	2.71	0.1183
14	10	1	-1	1	1	-1	4.24882	4.29	-0.0418
27	11	0	0	0	0	0	4.37059	4.52	-0.1448
3	12	-1	1	-1	-1	-1	4.1992	4.27	-0.0730
8	13	1	1	1	-1	-1	3.17529	3.26	-0.0862
28	14	0	0	0	0	0	4.38824	4.52	-0.1272
7	15	-1	1	1	-1	1	5.63541	5.71	-0.0795
11	16	-1	1	-1	1	1	5.97059	6.04	-0.0683

4	17	1	1	-1	-1	1	2.81	2.88	-0.0727
6	18	1	-1	1	-1	1	4.46	4.50	-0.0396
29	19	0	0	0	0	0	4.37059	4.52	-0.1448
12	20	1	1	-1	1	-1	2.836	2.91	-0.0749
20	21	0	2	0	0	0	3.46353	3.27	0.1975
21	22	0	0	-2	0	0	3.6118	3.52	0.0915
2	23	1	-1	-1	-1	-1	3.28	3.31	-0.0331
26	24	0	0	0	0	2	4.3647	4.26	0.1044
1	25	-1	-1	-1	-1	1	5.63529	5.66	-0.0265
15	26	-1	1	1	1	-1	5.6529	5.73	-0.0817
22	27	0	0	2	0	0	5.08471	4.95	0.1312
24	28	0	0	0	2	0	5.23529	5.13	0.1088
30	29	0	0	0	0	0	5	4.52	0.4846
17	30	-2	0	0	0	0	9.2753	9.17	0.1050
16	31	1	1	1	1	1	4.1588	4.24	-0.0814
10	32	1	-1	-1	1	1	3.70588	3.73	-0.0283

**Variable actual levels**																								
**X1: Working volume**					**X2: EDTA**					**X3: Inoculum size**					**X4: $CaCl_2$**					**X5: NaCl**				
-2	-1	0	1	2	-2	-1	0	1	2	-2	-1	0	1	2	-2	-1	0	1	2	-2	-1	0	1	2
300	400	500	600	700	0.0005	0.001	0.002	0.003	0.004	3%	5%	7%	9%	11%	0.026	0.036	0.046	0.056	0.066	10	15	20	25	30

Table S4. FCCD regression statistics, regression coefficients of second order polynomial model for cyanobacterial polysaccharide production.

Factor	Coefficient Estimate	Standard Error	95% CI Low	95% CI High
Intercept	4.52	0.1135	4.27	4.77
A-Working volume	-1.01	0.0581	-1.13	-0.8780
B-EDTA	-0.2654	0.0581	-0.3933	-0.1376
C-Inoculum size	0.3583	0.0581	0.2305	0.4861
D-$CaCl_2$	0.3483	0.0581	0.2205	0.4762
E-NaCl	0.3888	0.0581	0.2610	0.5166
AB	-0.0523	0.0711	-0.2089	0.1042
AC	0.0731	0.0711	-0.0835	0.2296
AD	-0.1960	0.0711	-0.3525	-0.0394
AE	-0.1912	0.0711	-0.3478	-0.0347
BC	-0.0025	0.0711	-0.1590	0.1541
BD	0.0008	0.0711	-0.1557	0.1574
BE	-0.0516	0.0711	-0.2082	0.1050
CD	0.0917	0.0711	-0.0649	0.2482
CE	0.0985	0.0711	-0.0580	0.2551
DE	-0.0019	0.0711	-0.1584	0.1547
A^2	0.6608	0.0525	0.5452	0.7764
B^2	-0.1796	0.0525	-0.2953	-0.0640
C^2	-0.0696	0.0525	-0.1852	0.0460
D^2	-0.0214	0.0525	-0.1370	0.0942
E^2	-0.2582	0.0525	-0.3738	-0.1425
**Std. Dev.**	0.2845		**R^2**	0.9839
**Mean**	4.61		**Adjusted R^2**	0.9547
**C.V. %**	6.17		**Predicted R^2**	0.9060
**PRESS**	5.76		**Adeq Precision**	28.0502

Table S5. Analysis of variance (ANOVA) for the quadratic regression model obtained from FCCD for cyanobacterial polysaccharide production.

Source	Sum of Squares	Df[1]	Mean Square	F-value[2]	p-value[3]
**Model**	54.53	20	2.73	33.68	< 0.0001
A-Working volume	24.28	1	24.28	299.95	< 0.0001
B-EDTA	1.69	1	1.69	20.89	0.0008
C-Inoculum size	3.08	1	3.08	38.06	< 0.0001
D-$CaCl_2$	2.91	1	2.91	35.98	< 0.0001
E-NaCl	3.63	1	3.63	44.81	< 0.0001
AB	0.0438	1	0.0438	0.5414	0.4772
AC	0.0854	1	0.0854	1.06	0.3264
AD	0.6144	1	0.6144	7.59	0.0187
AE	0.5850	1	0.5850	7.23	0.0211
BC	0.0001	1	0.0001	0.0012	0.9727
BD	0.0000	1	0.0000	0.0001	0.9910
BE	0.0426	1	0.0426	0.5263	0.4833
CD	0.1344	1	0.1344	1.66	0.2240
CE	0.1553	1	0.1553	1.92	0.1934
DE	0.0001	1	0.0001	0.0007	0.9795
A^2	12.81	1	12.81	158.24	< 0.0001
B^2	0.9465	1	0.9465	11.69	0.0057
C^2	0.1422	1	0.1422	1.76	0.2120
D^2	0.0134	1	0.0134	0.1658	0.6917
E^2	1.95	1	1.95	24.15	0.0005
**Residual**	0.8904	11	0.0809		
**Lack of Fit**	0.2055	6	0.0343	0.2501	0.9392
**Pure Error**	0.6849	5	0.1370		
**Cor. Total**	55.42	31			

(1) Df: Degree of freedom, (2) F: Fishers's function, (3) P: Level of significance

Table S6. Displayed the fit summary of FCCD for cyanobacterial EPS production.

**Sequential model Sum of Squares**					
**Source**	**Sum of Squares**	**Df**	**Mean Square**	**F-value**	**p-value**
Mean vs Total	681.37	1	681.37		
Linear vs Mean	35.59	5	7.12	9.34	< 0.0001
2FI vs Linear	1.66	10	0.1661	0.1463	0.9980
**Quadratic vs 2FI**	**17.27**	**5**	**3.45**	**42.68**	**< 0.0001**
Cubic vs Quadratic	0.0238	5	0.0048	0.0329	0.9991
Residual	0.8667	6	0.1444		
**Lack of Fit Tests**					
**Source**	**Sum of Squares**	**Df**	**Mean Square**	**F-value**	**p-value**
Linear	19.14	21	0.9114	6.65	0.0223
2FI	17.48	11	1.59	11.60	0.0071
**Quadratic**	**0.2055**	**6**	**0.0343**	**0.2501**	**0.9392**
Cubic	0.1818	1	0.1818	1.33	0.3014
Pure Error	0.6849	5	0.1370		
**Model Summary Statistics**					
**Source**	**Std. Dev.**	**R^2**	**Adjusted R^2**	**Predicted R^2**	**PRESS**
Linear	0.8732	0.6423	0.5735	0.4456	30.72
2FI	1.07	0.6722	0.3649	0.4821	28.70
**Quadratic**	**0.2845**	**0.9839**	**0.9547**	**0.9060**	**5.76**
